# Supplementary material for: Promising Oxygen- and Nitrogen-Rich Azidonitramino Ether Plasticizers for Energetic Materials
Source: Molecules. 2022 Nov 10;27(22):7749. doi: 10.3390/molecules27227749 (PMC9695530; doi:10.3390/molecules27227749)
Supplement: Supplementary file 1 [file molecules-27-07749-s001.zip › molecules-2006872-supplementary.pdf]

Supplementary Materials

Promising oxygen- and nitrogen-rich azidonitramino ether plasticizers for energetic materials

Dmitry B. Vinogradov [1], Pavel V. Bulatov [1], Evgeny Yu. Petrov [1], Pavel S. Gribov [1], Natalia N. Kondakova [2], Natalia N. Il′icheva [2], Evgenia R. Stepanova [2], Anatoly P. Denisyuk [2], Vladimir A. Sizov [2], Valery P. Sinditskii [2] and Aleksei B. Sheremetev [1,*]

[1] Zelinsky Institute of Organic Chemistry, Russian Academy of Sciences, 47 Leninsky prosp., 119991 Moscow, Russia
[2] Mendeleev University of Chemical Technology, 9 Miusskaya pl., 125047 Moscow, Russia
* Correspondence: sab@ioc.ac.ru

Table of Contents

Decomposition in non-isothermal conditions

Decomposition in isothermal conditions

Relaxation and phase transitions

NMR spectra

Caution! Although we have encountered no difficulties during preparation and handling of these compounds, they are potentially explosive energetic materials. Manipulations must be carried out by using appropriate standard safety precautions.

Decomposition in non-isothermal conditions

Differential scanning calorimetry (DSC) and thermogravimetric analysis (TGA) were performed with a DSC 822e Mettler Toledo in the temperature range of 25-400 °C at 10 K per min heating rates. The samples weighing between 1 mg and 2 mg were analyzed in closed aluminum pans with pierced lids under dynamic nitrogen atmosphere. Examples of the obtained DSC and TGA curves are shown in Figures S1–S8.

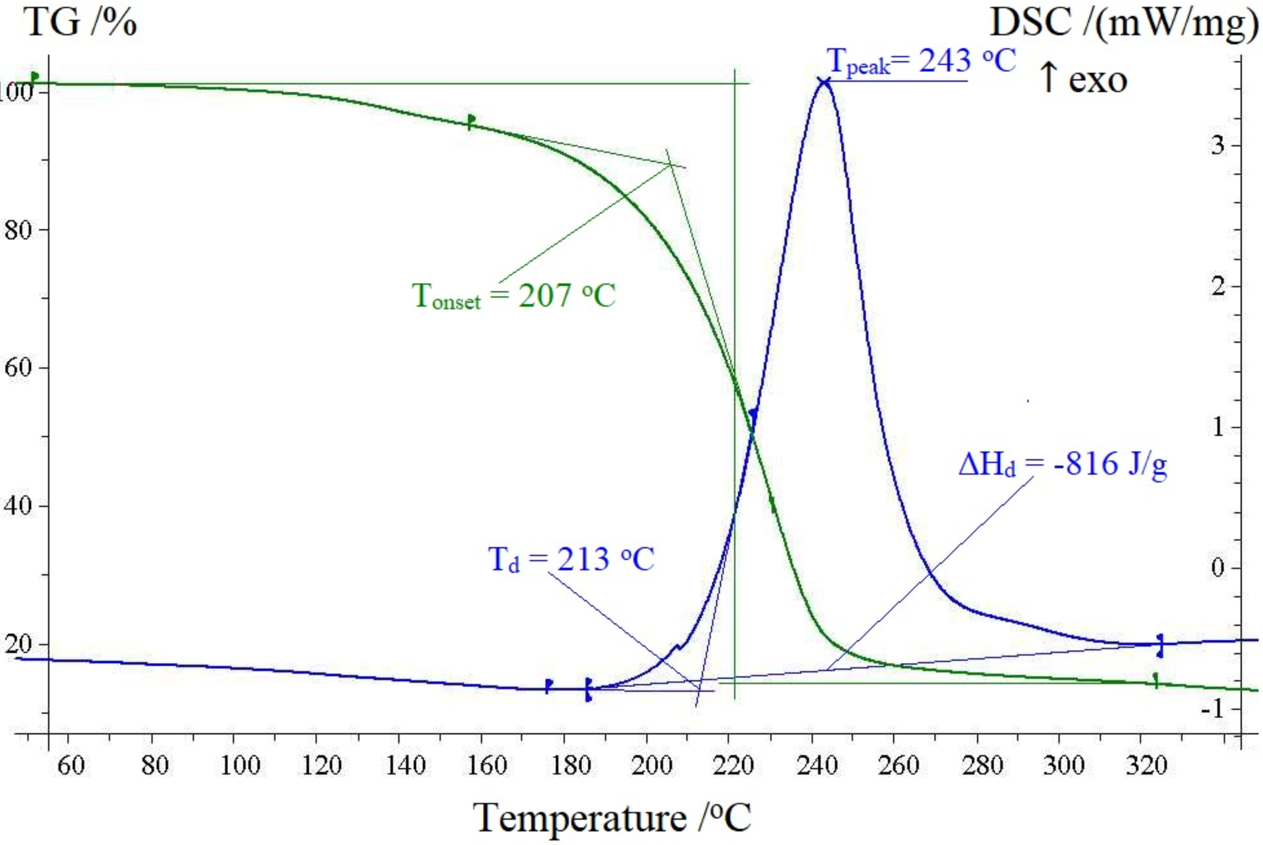

Figure S1. Thermograms of compound **3a**: blue line is DSC; green line is TGA.

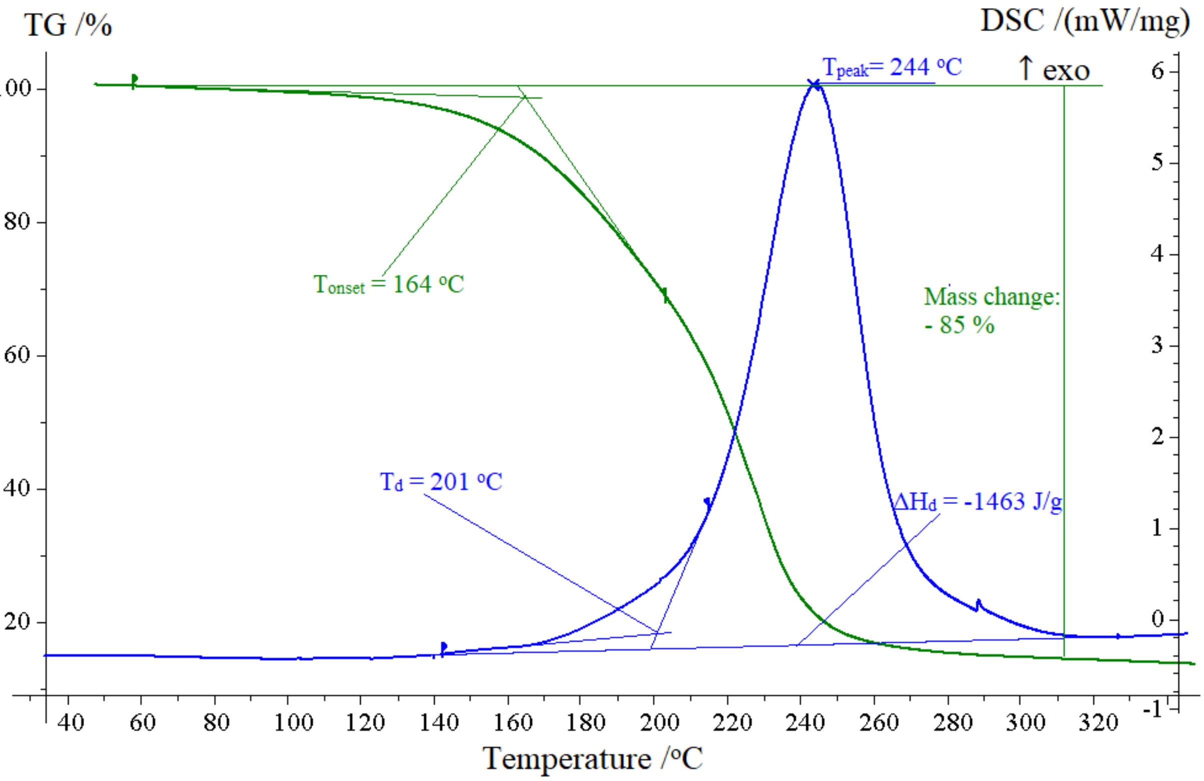

Figure S2. Thermograms of compound **3b**: blue line is DSC; green line is TGA.

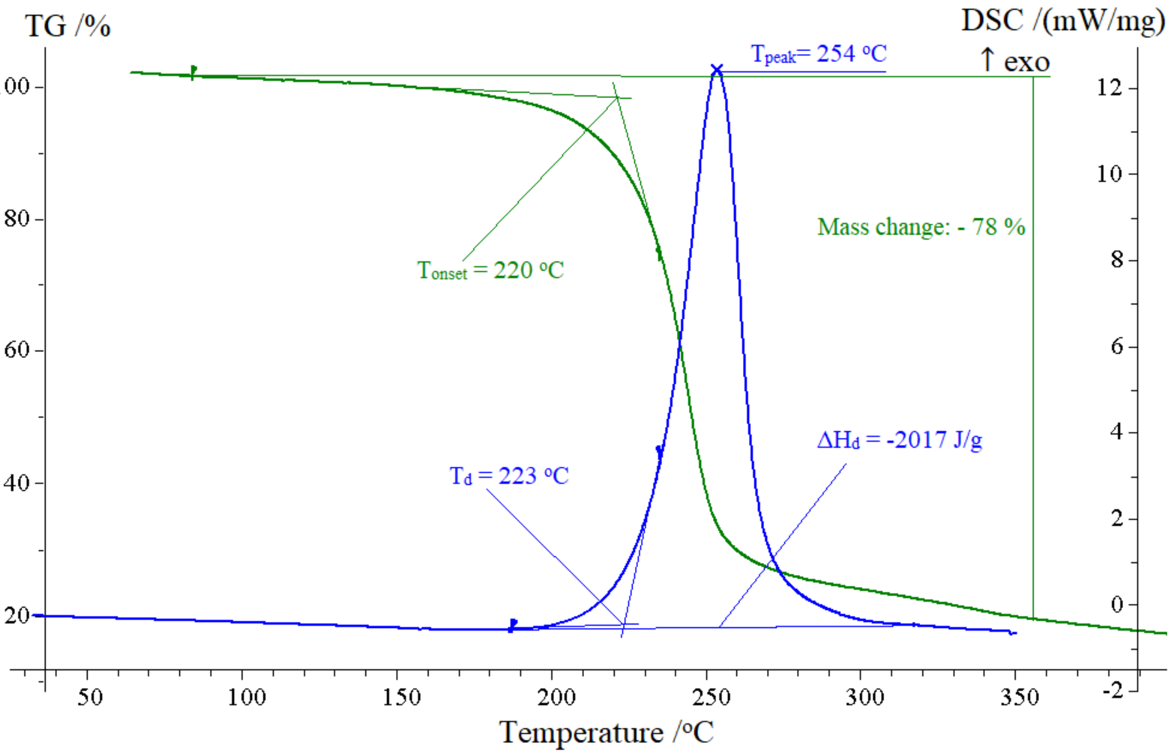

Figure S3. Thermograms of compound **3c**: blue line is DSC; green line is TGA.

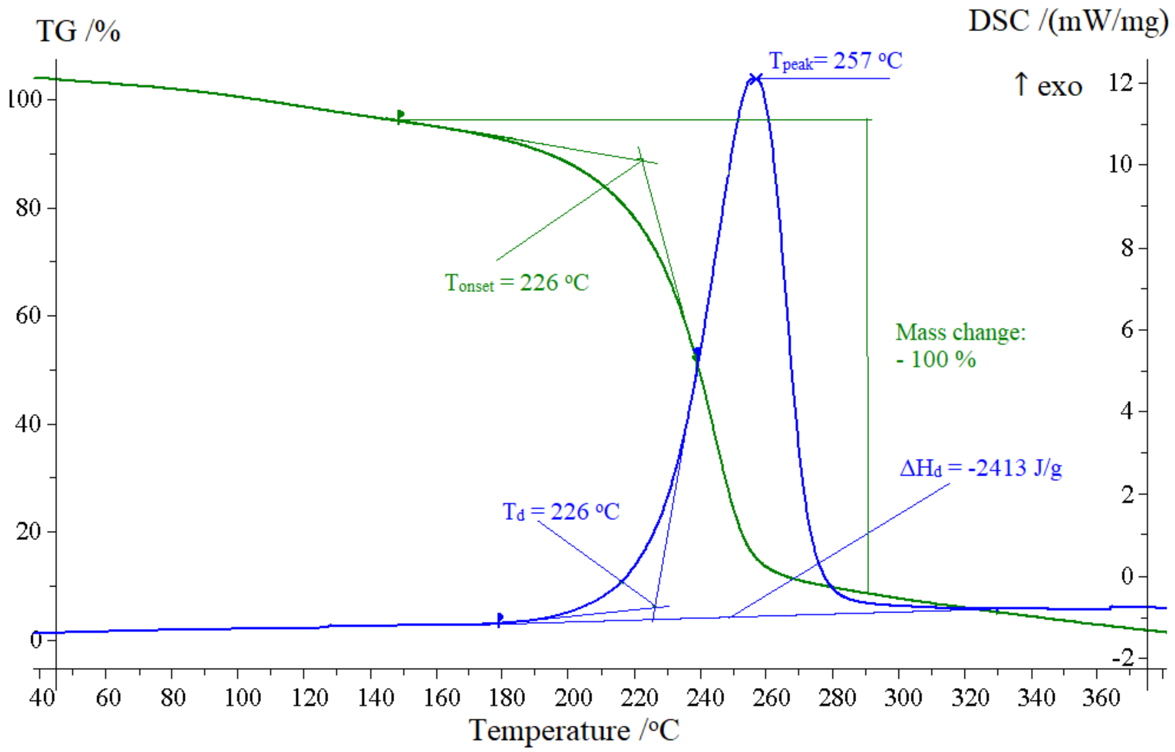

Figure S4. Thermograms of compound **3d**: blue line is DSC; green line is TGA.

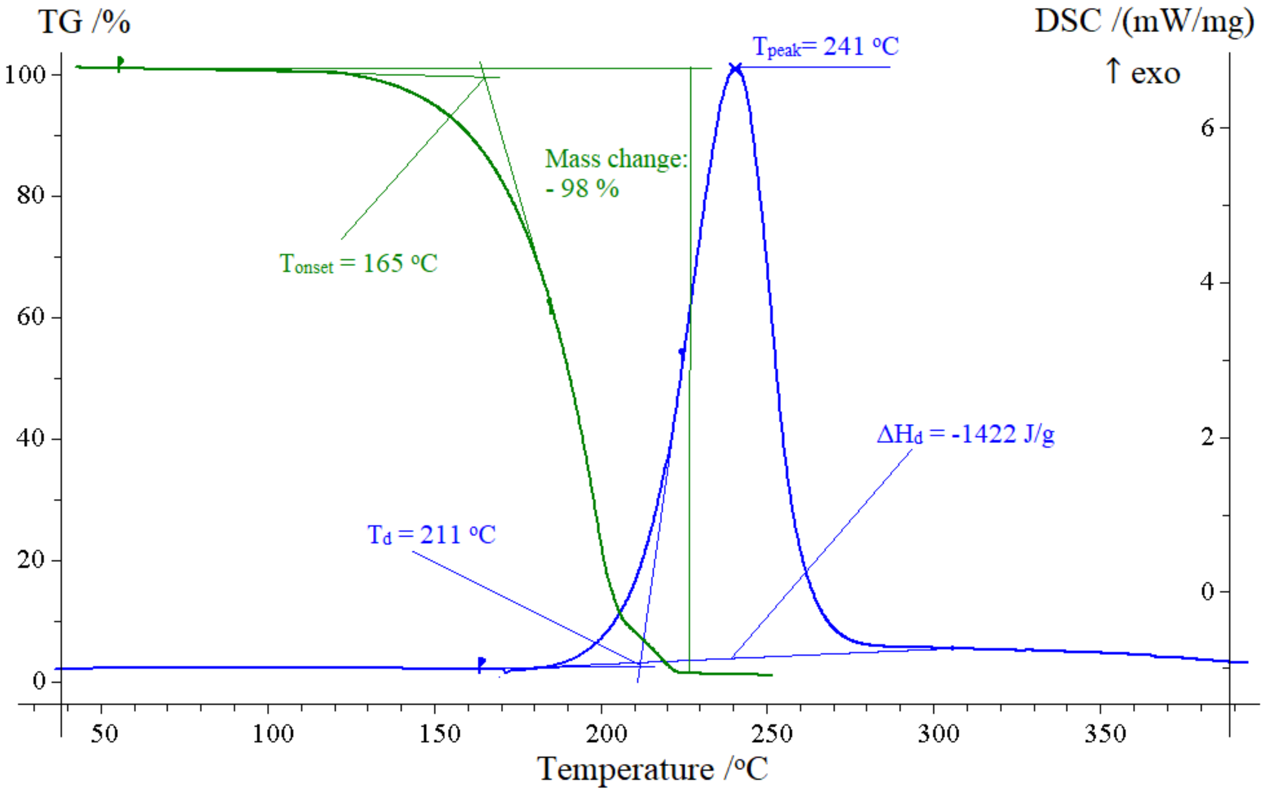

Figure S5. Thermograms of compound **6**: blue line is DSC; green line is TGA.

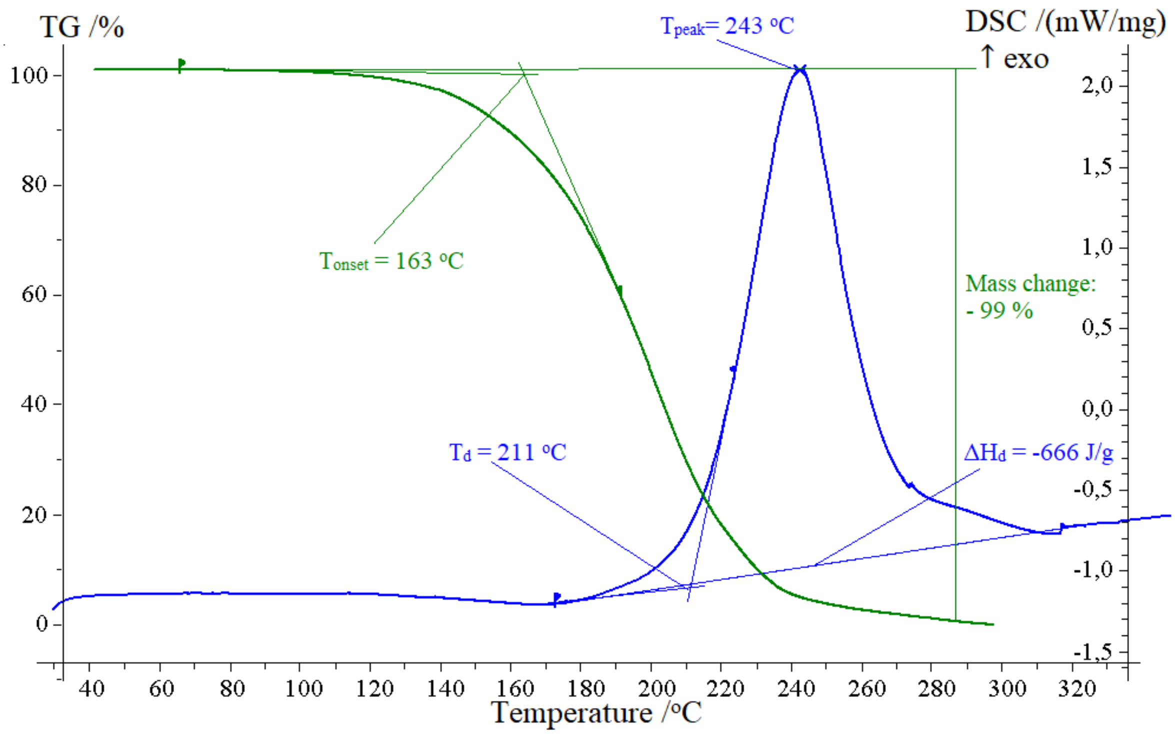

Figure S6. Thermograms of compound **10**: blue line is DSC; green line is TGA.

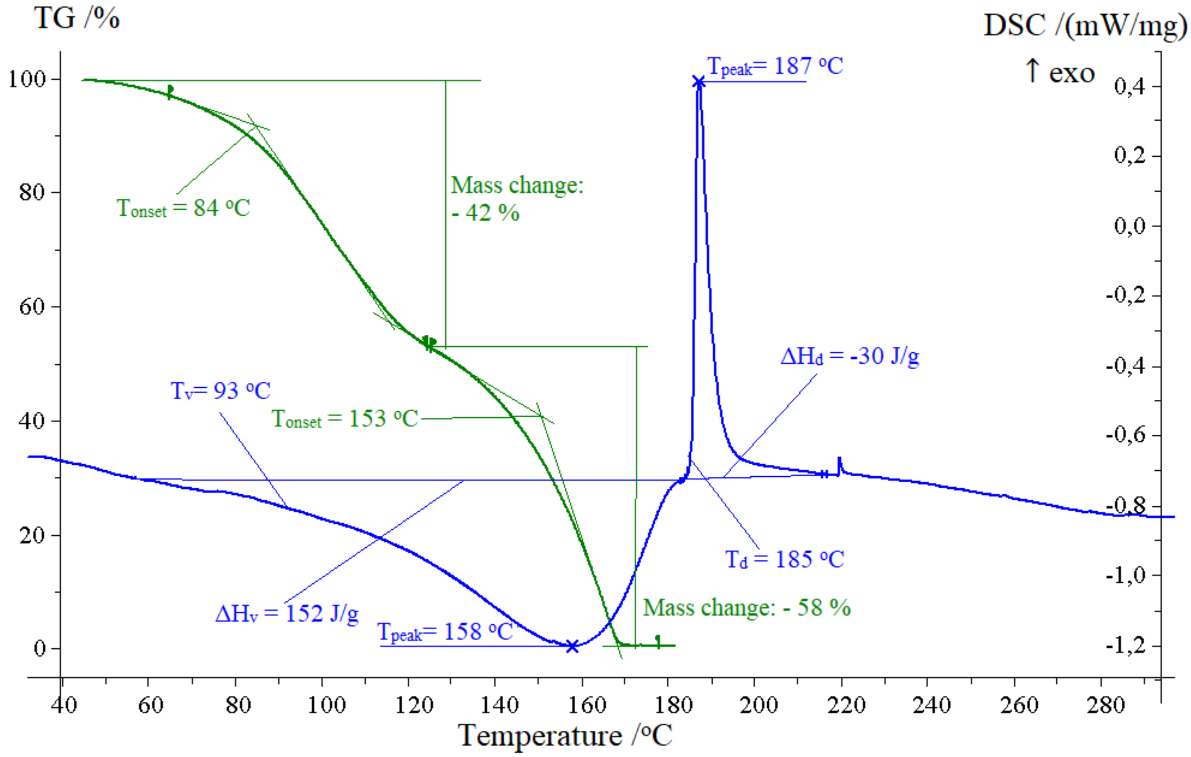

Figure S7. Thermograms of compound **13**: blue line is DSC; green line is TGA.

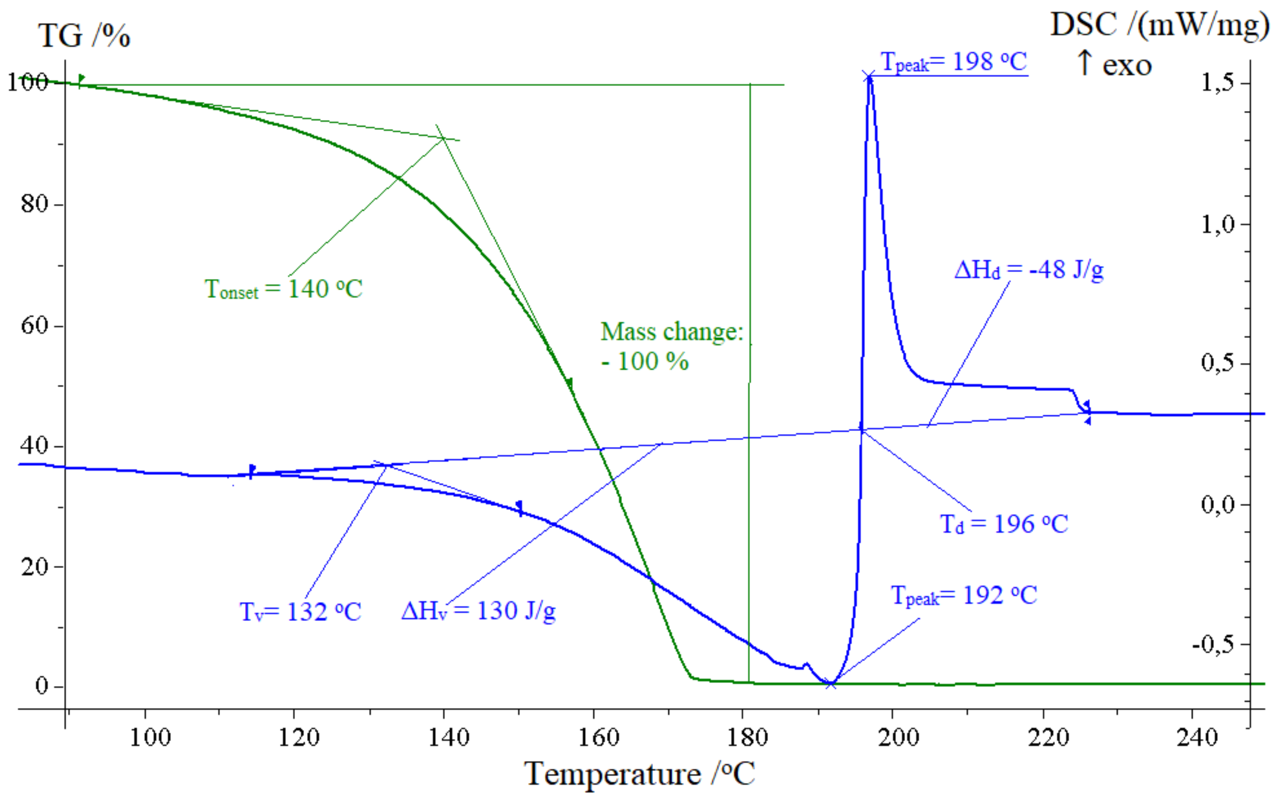

Figure S8. Thermograms of **NG**: blue line is DSC; green line is TGA.

Decomposition in isothermal conditions

The experiments on the decomposition of compounds **3a**, **3c** and **6** under isothermal conditions were carried out in thin-walled glass manometers of the compensation type (the Bourdon glass gauge). Samples of about 20-60 mg weight were loaded into a glass manometer with a volume of 10-12 cm^3. The ampules were vacuumed to 0.1 Torr, sealed, and put in a thermostat with the Wood's alloy. Pressure of gases evolved in the decomposition experiments (the accuracy of pressure measurements was ±1 mm Hg) was converted to the gas volume (V) at normal conditions. Examples of the obtained V vs. time curves are shown in Figures S9–S11.

The description of the experimental dependence of gas release on time V(t) by a suitable model allows one to obtain the rate constants. The rate constants of compounds **3a** and **6** were calculated using the first order decomposition equation:

$$V = V_{end}\cdot(1-\exp(-k\cdot t)),$$

where V_{end} is the final volume of evolved gases.

The rate constant of compound **3c** was calculated using the model of first-order reaction with self-acceleration:

$$V = V_{end} \cdot k_1(\exp((k_1+k_2)\cdot t)-1)/(k_2 + k_1 \cdot \exp((k_1+k_2)\cdot t)),$$

where k_1 is rate constant of non-catalytic stage, k_2 is pseudo first-order rate constant of catalytic stage, and t is time.

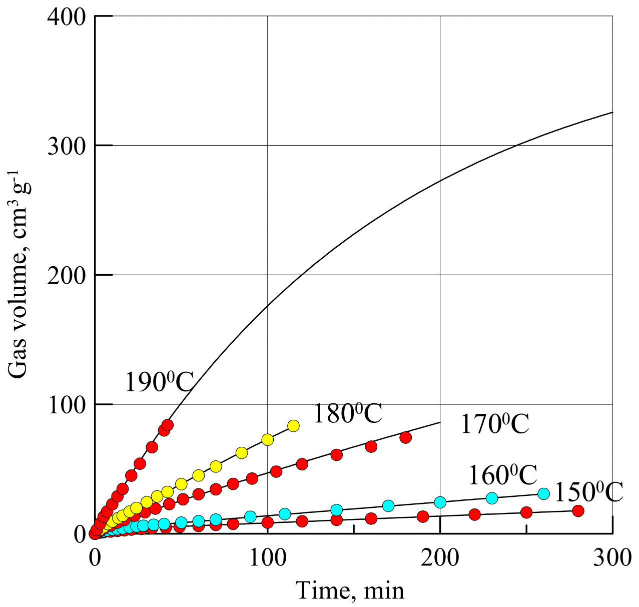

Figure S9. Gas release curves of compound **3a** at different temperatures. Points are experiment, lines are fittings.

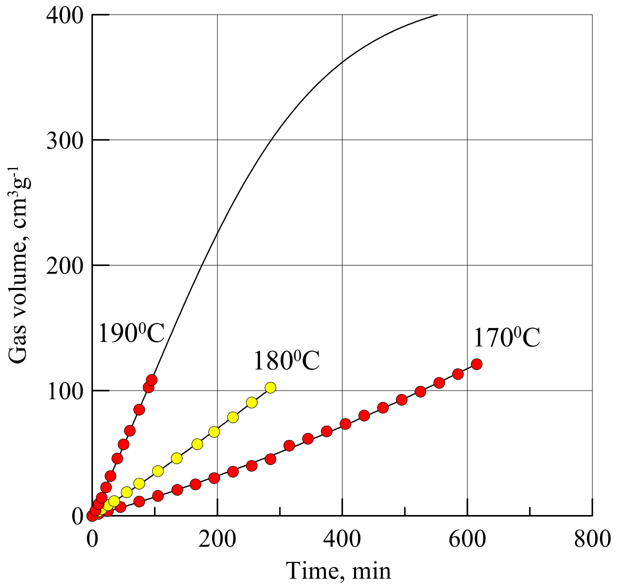

Figure S10. Gas release curves of compound **3c** at different temperatures. Points are experiment, lines are fittings.

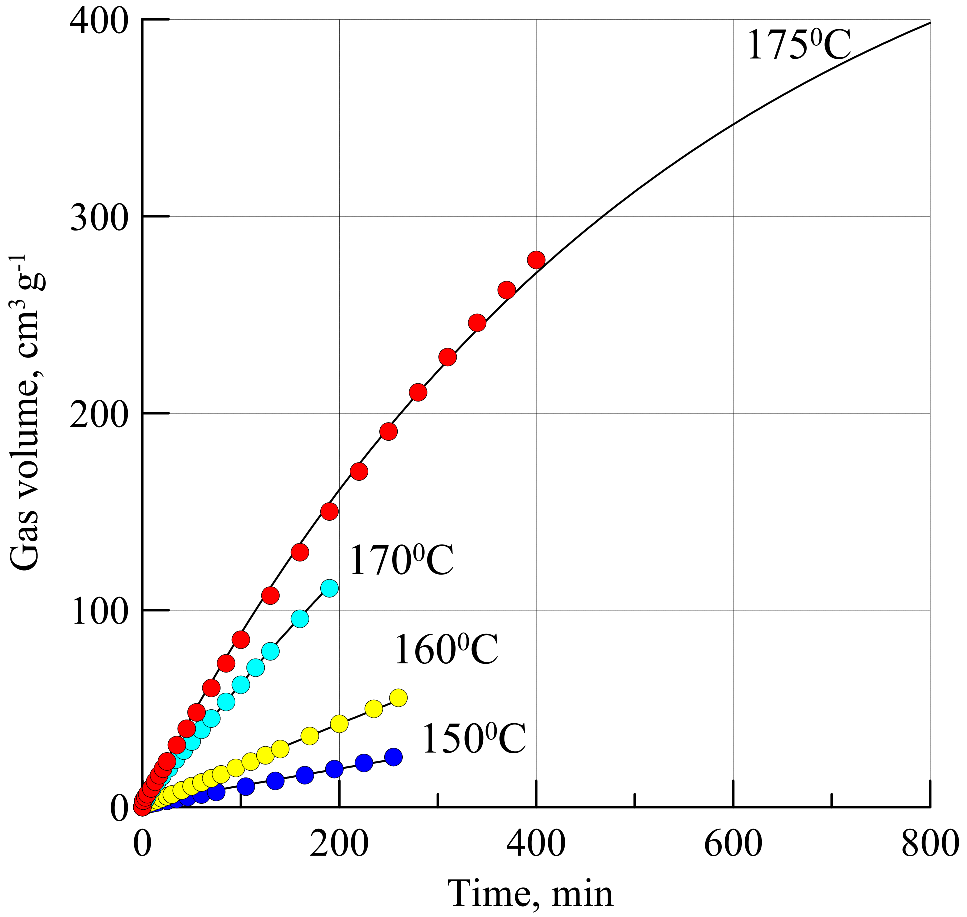

Figure S11. Gas release curves of compound **6** at different temperatures. Points are experiment, lines are fittings.

Relaxation and phase transitions

The study of relaxation and phase transitions was carried out by the method of differential scanning calorimetry. During the experiment, the samples were placed in a 40 μl aluminum cuvette, cooled uncontrollably, and then heated at a rate of 10 degrees per minute. The typical thermograms are presented in Figures S12–S19.

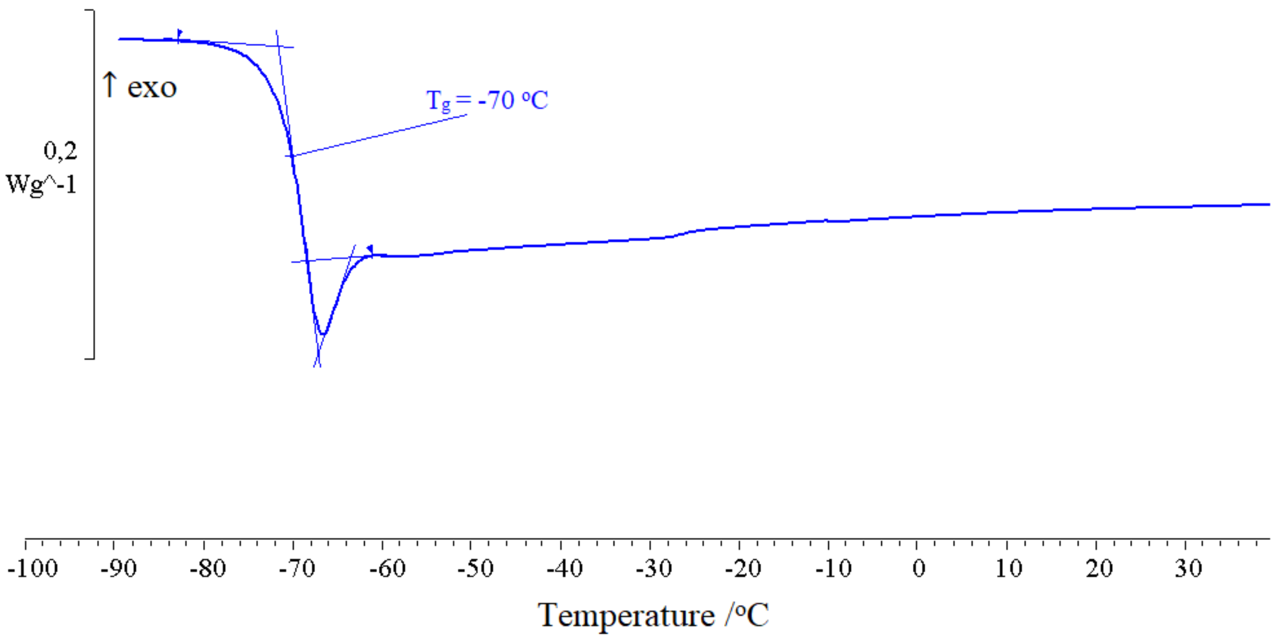

Figure S12. Thermograms of **NG**. Heating rate 10 deg/min.

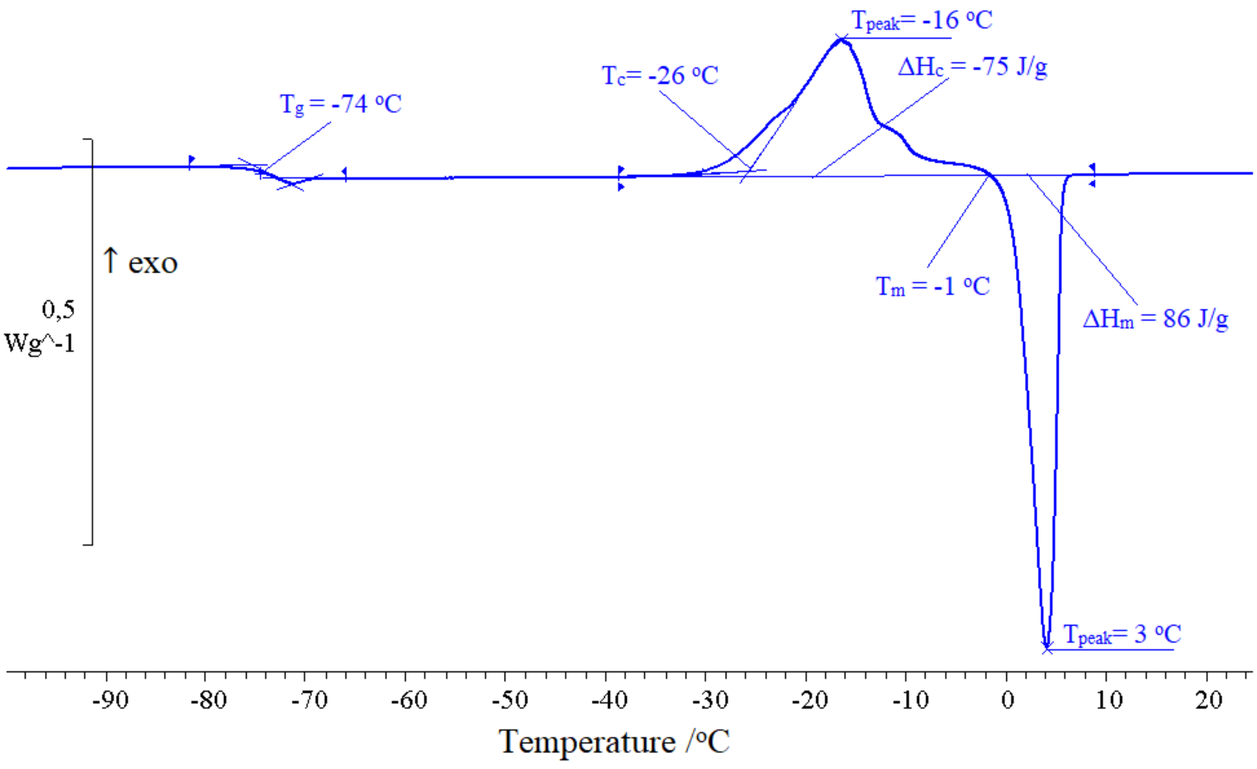

Figure S13. Thermograms of **NG**. Heating rate 1.25 deg/min.

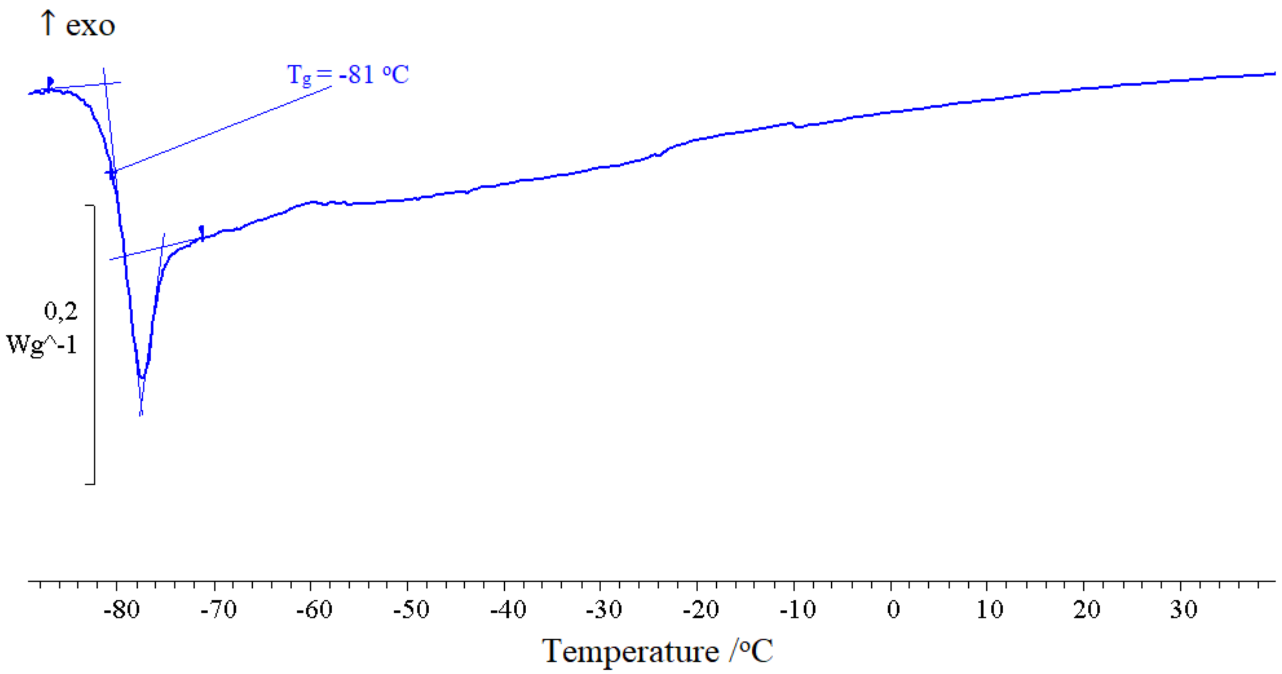

Figure S14. Thermograms of compound **3a**. Heating rate 10 deg/min.

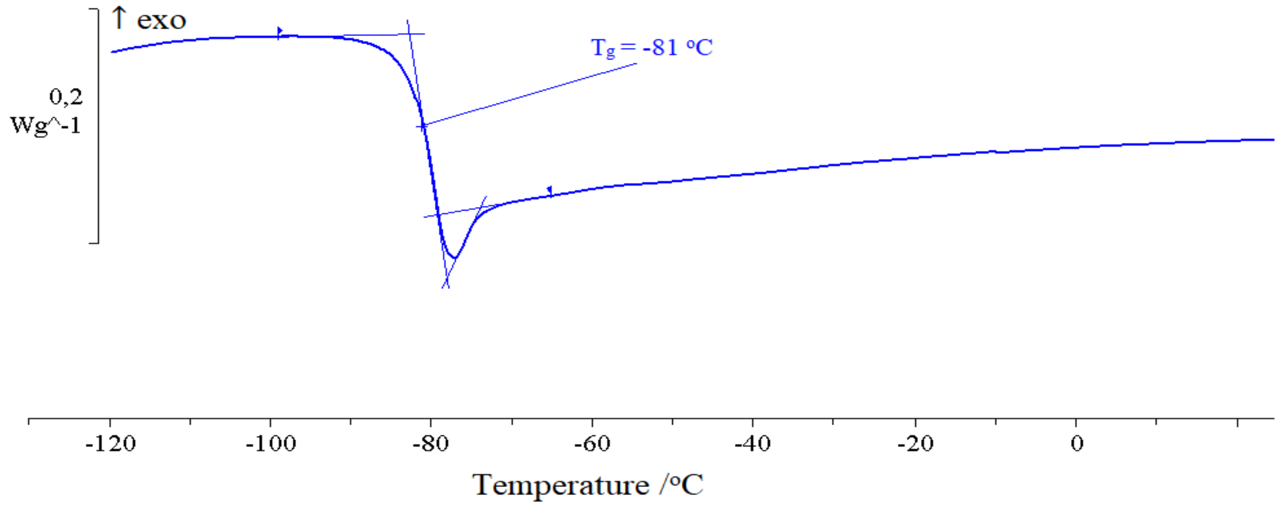

Figure S15. Thermograms of compound **3b**. Heating rate 10 deg/min.

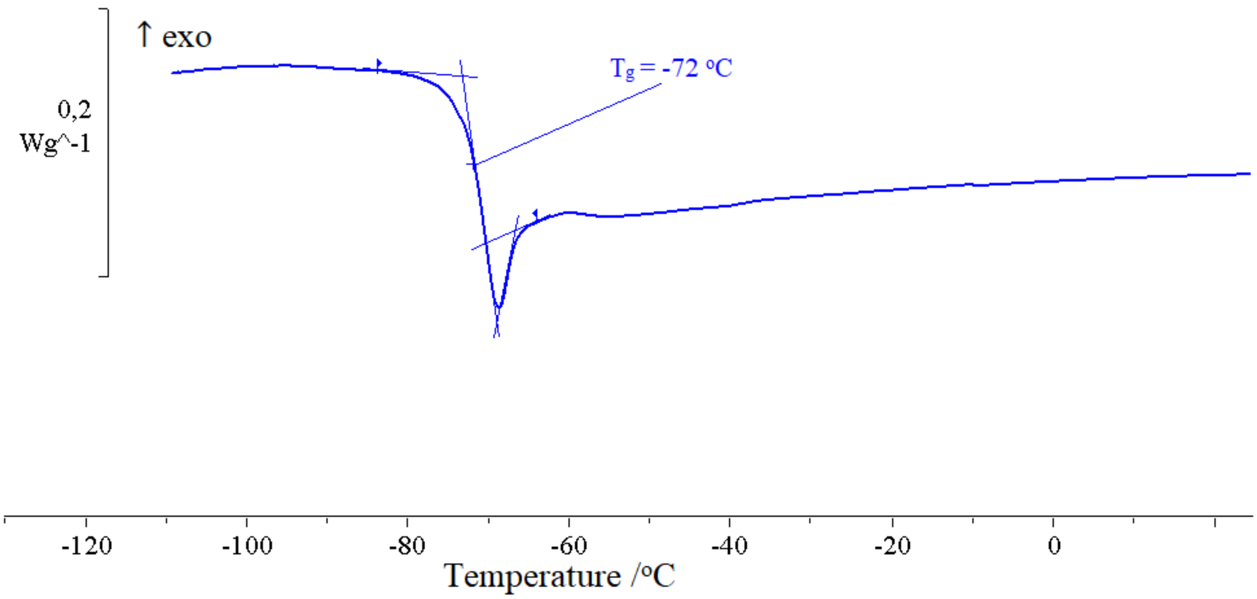

Figure S16. Thermograms of compound **3c**. Heating rate 10 deg/min.

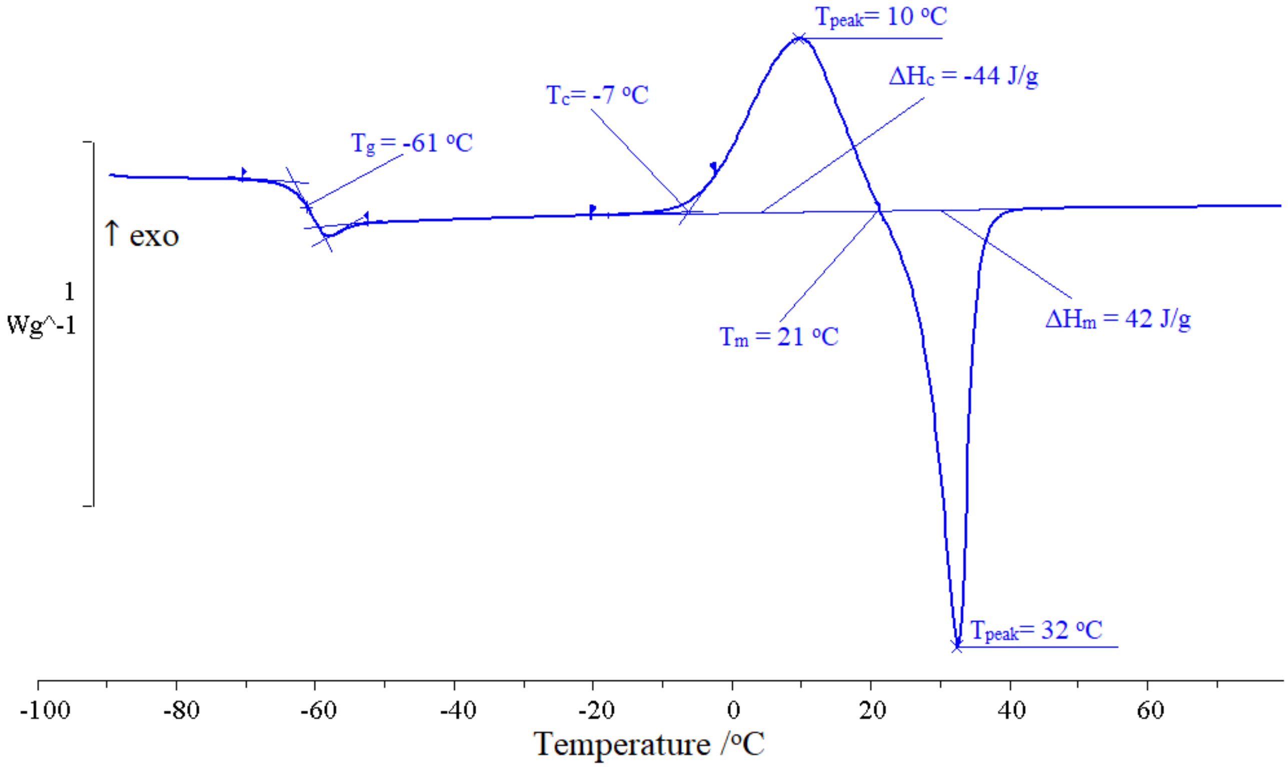

Figure S17. Thermograms of compound **3d**. Heating rate 10 deg/min.

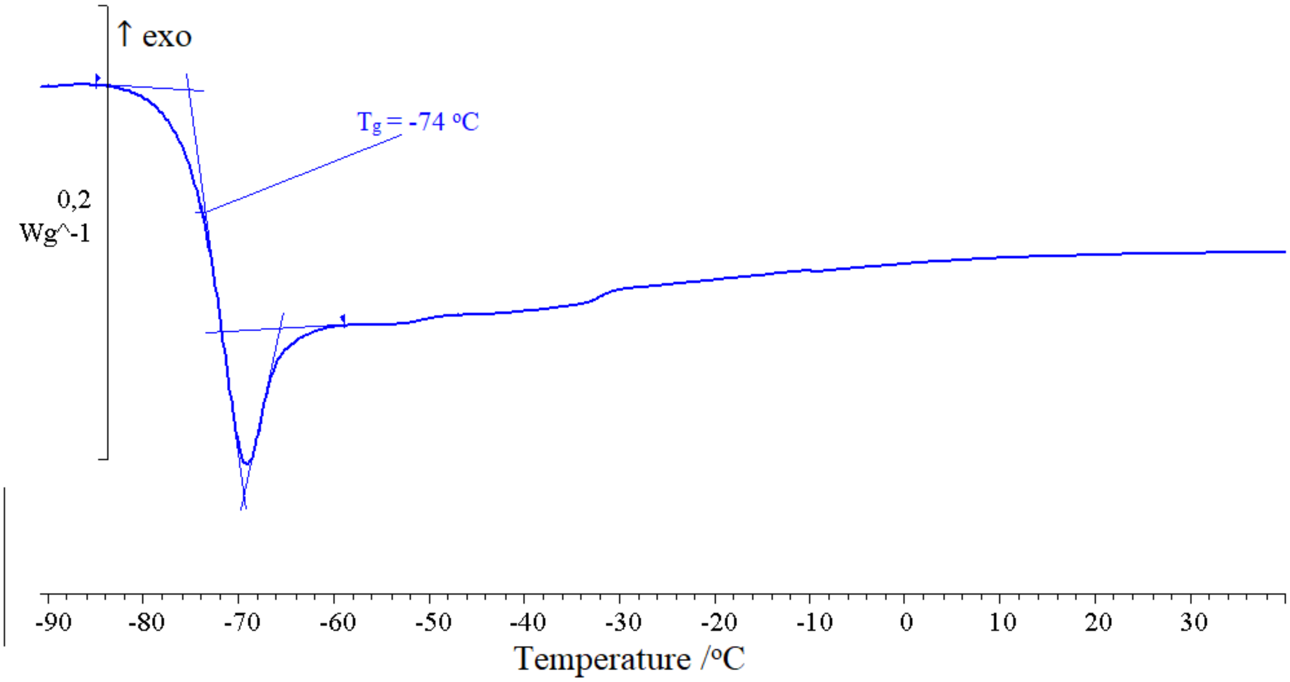

Figure S18. Thermograms of compound **10.** Heating rate 10 deg/min.

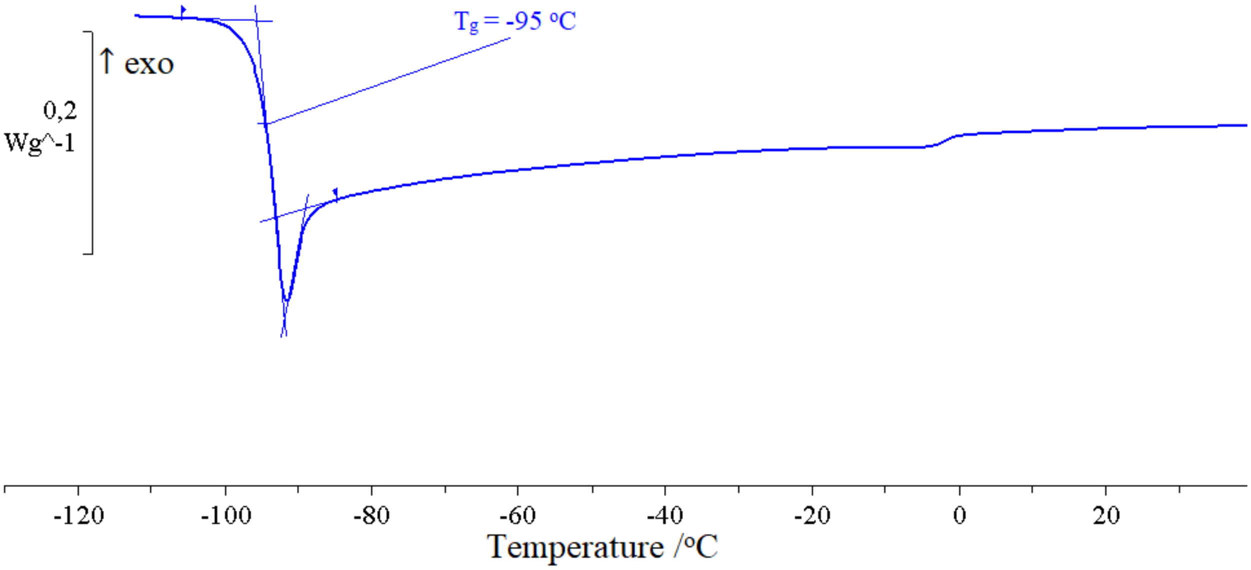

Fugure S19. Thermograms of compound **13**. Heating rate 10 deg/min.

^{1}H NMR in DMSO-d6

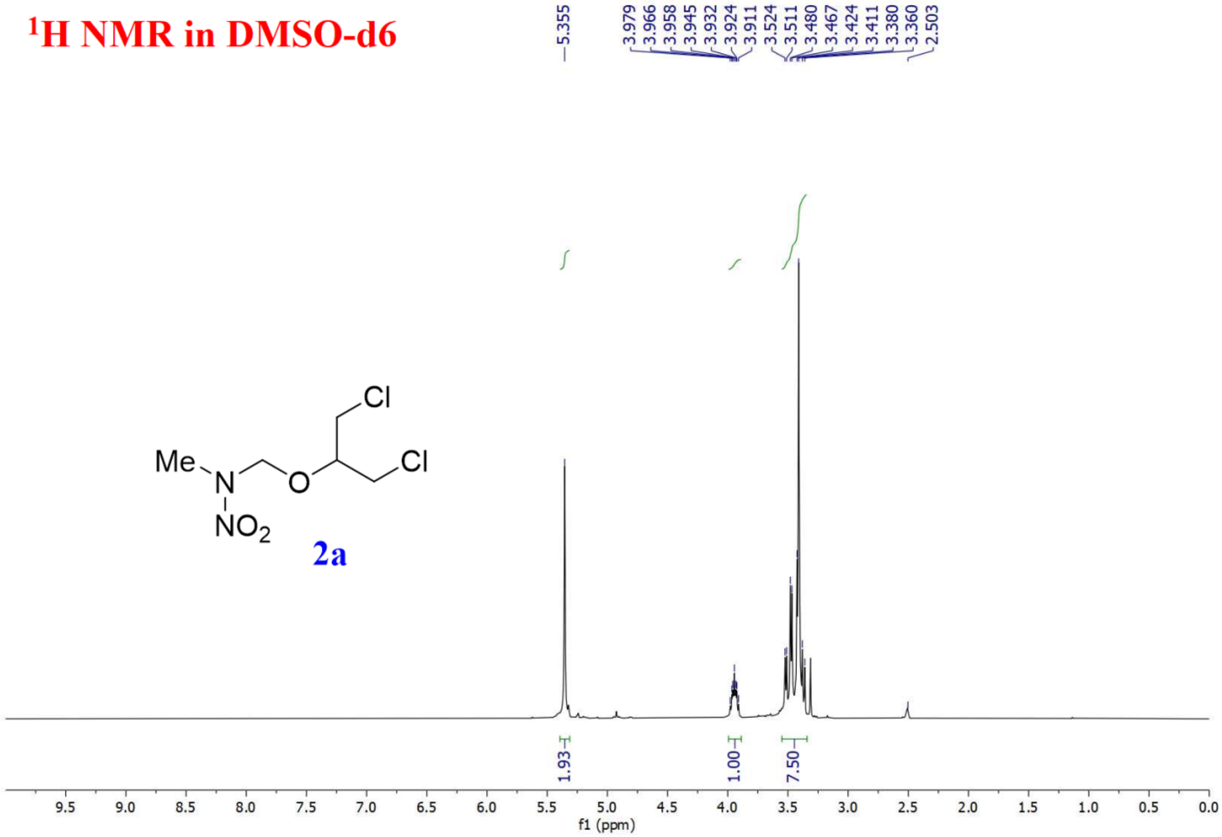

^{13}C NMR in DMSO-d6

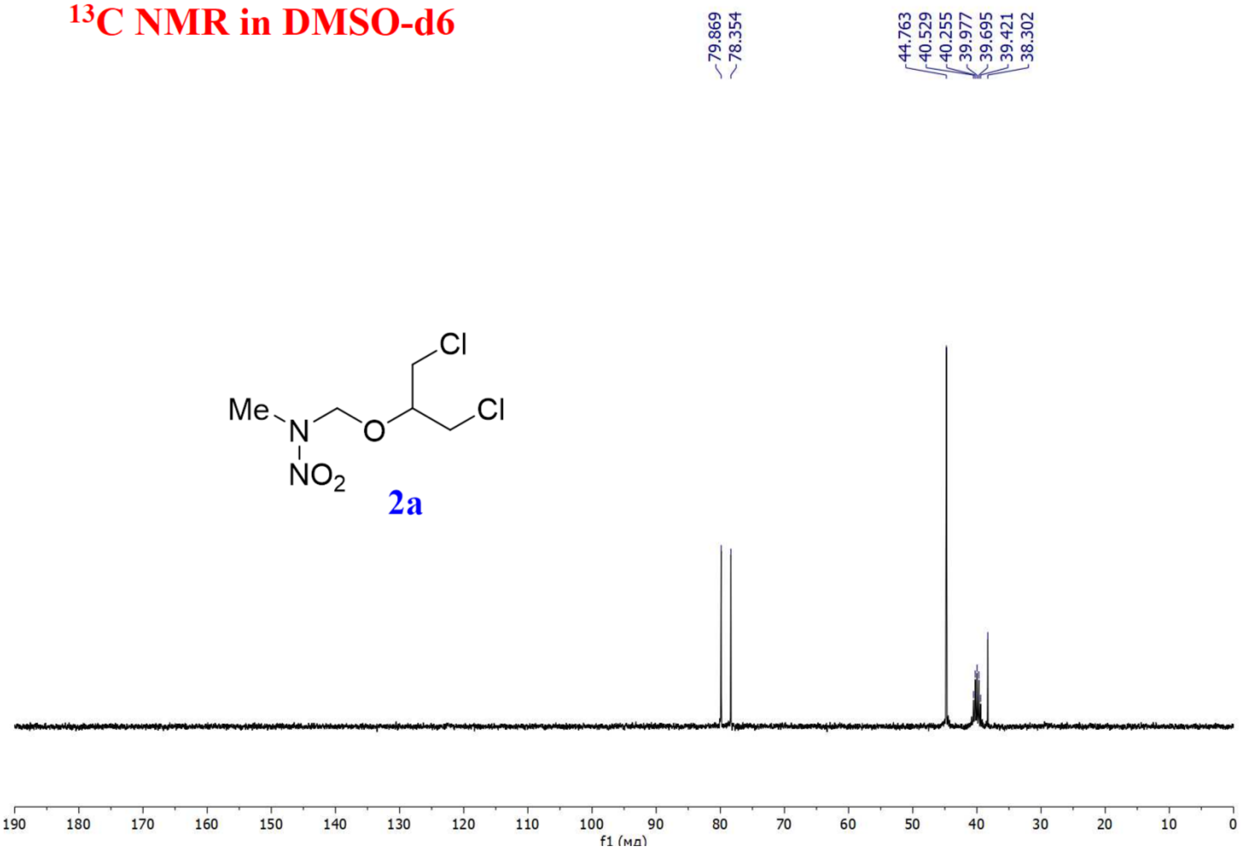

^{1}H NMR in DMSO-d6

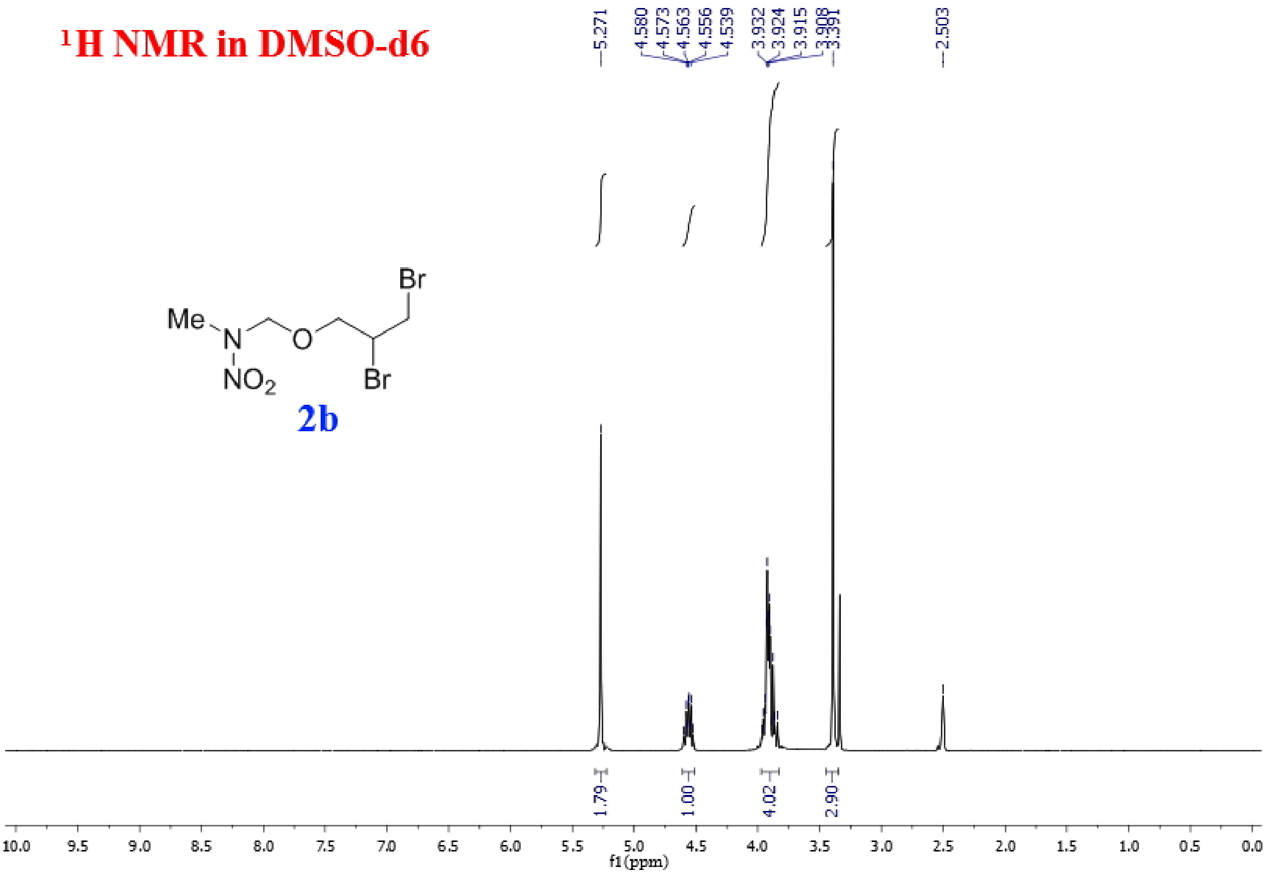

^{13}C NMR in DMSO-d6

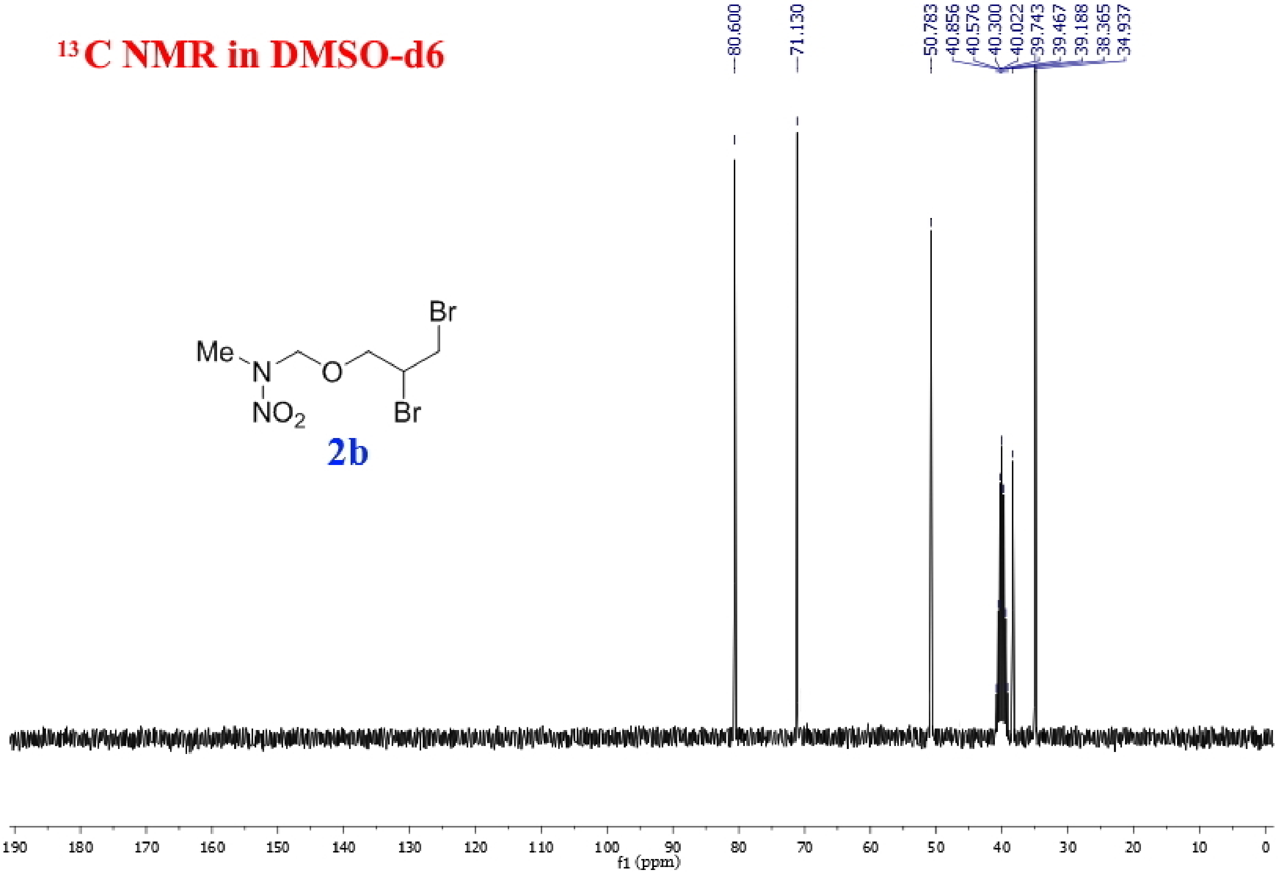

2b

^{1}H NMR in DMSO-d6

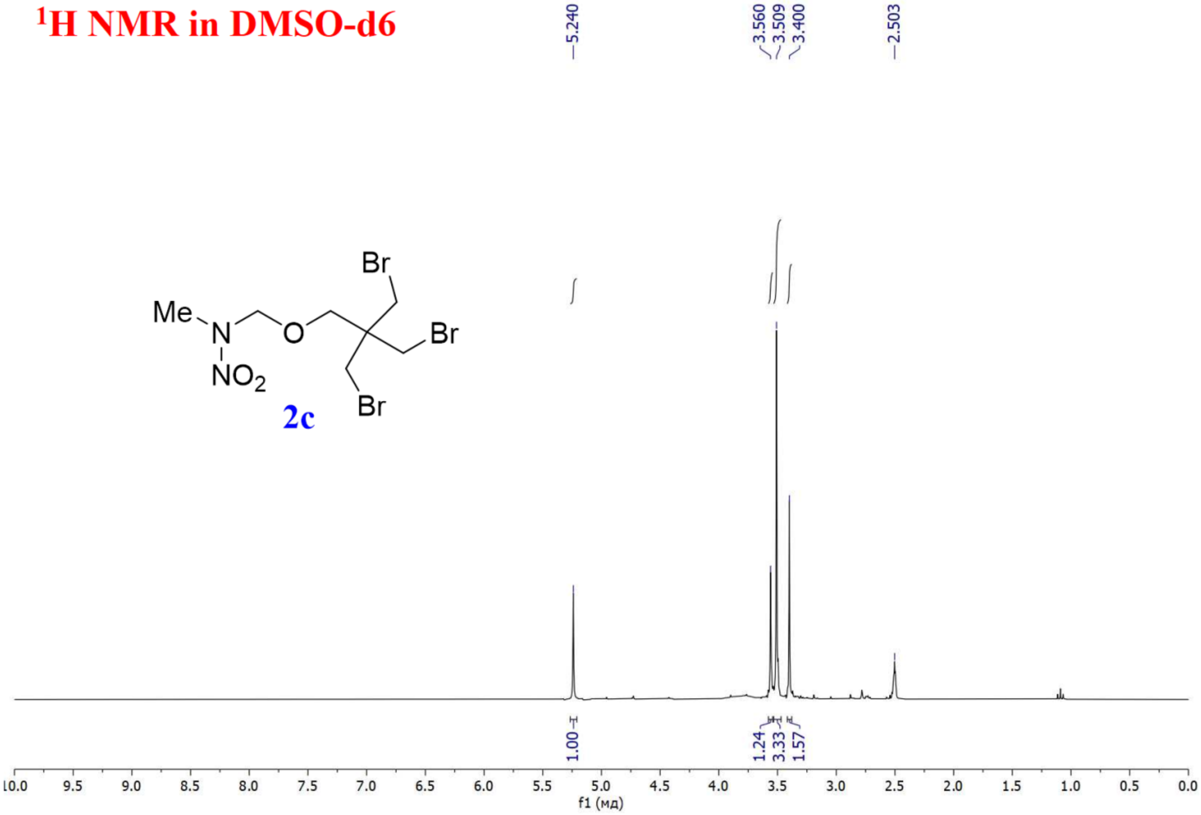

^{13}C NMR in DMSO-d6

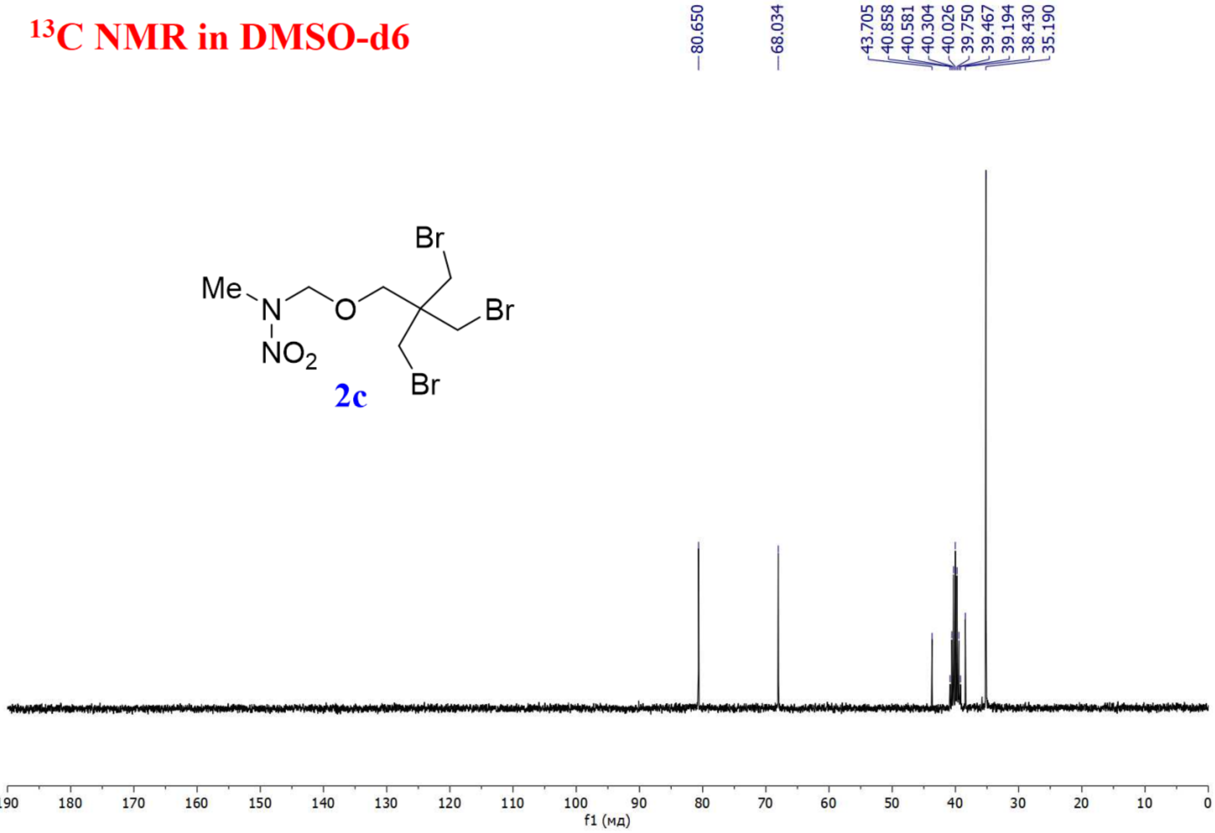

^{1}H NMR in DMSO-d6

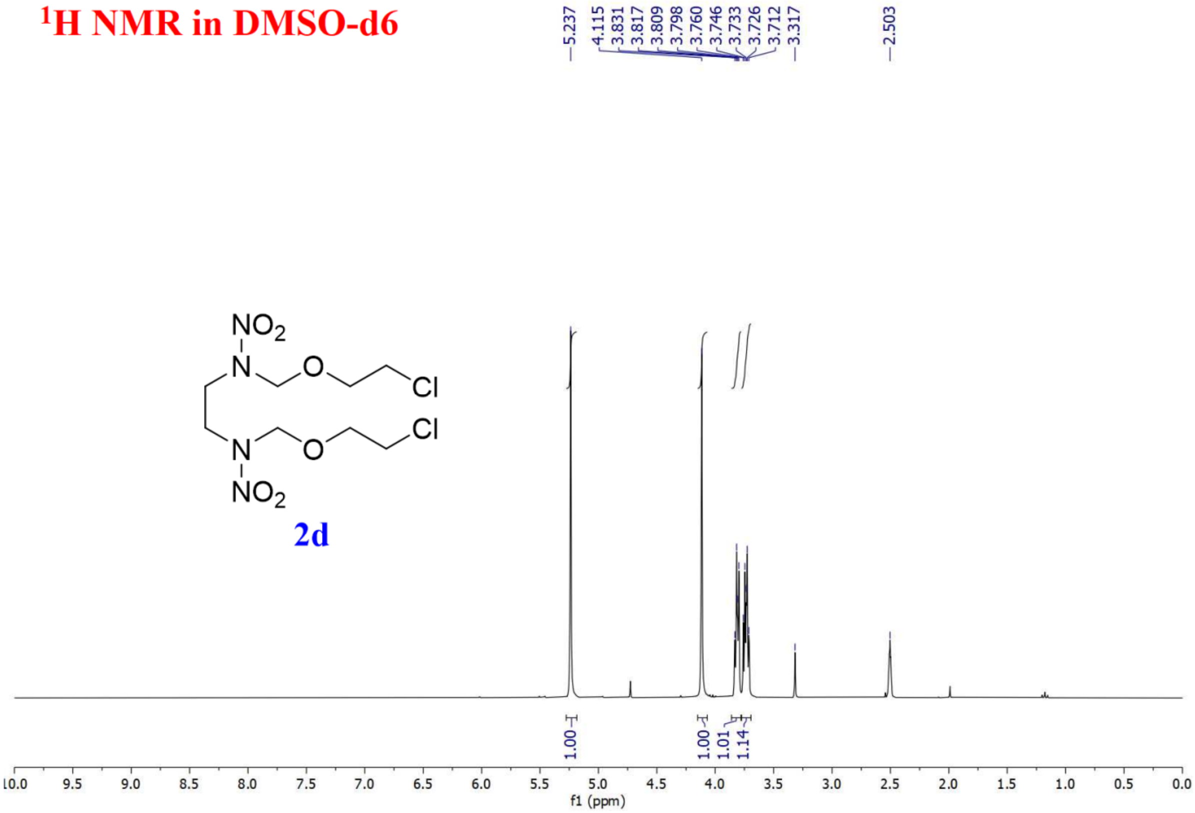

^{13}C NMR in DMSO-d6

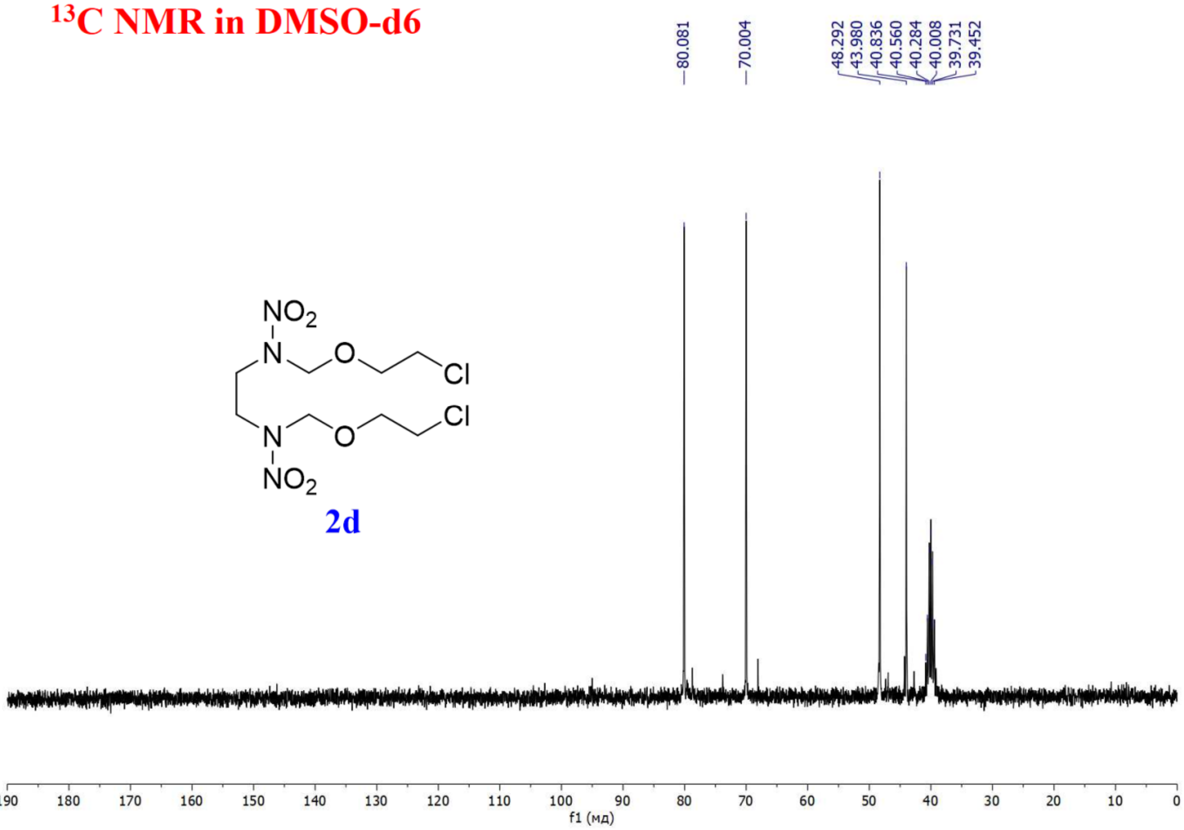

^{1}H NMR in DMSO-d6

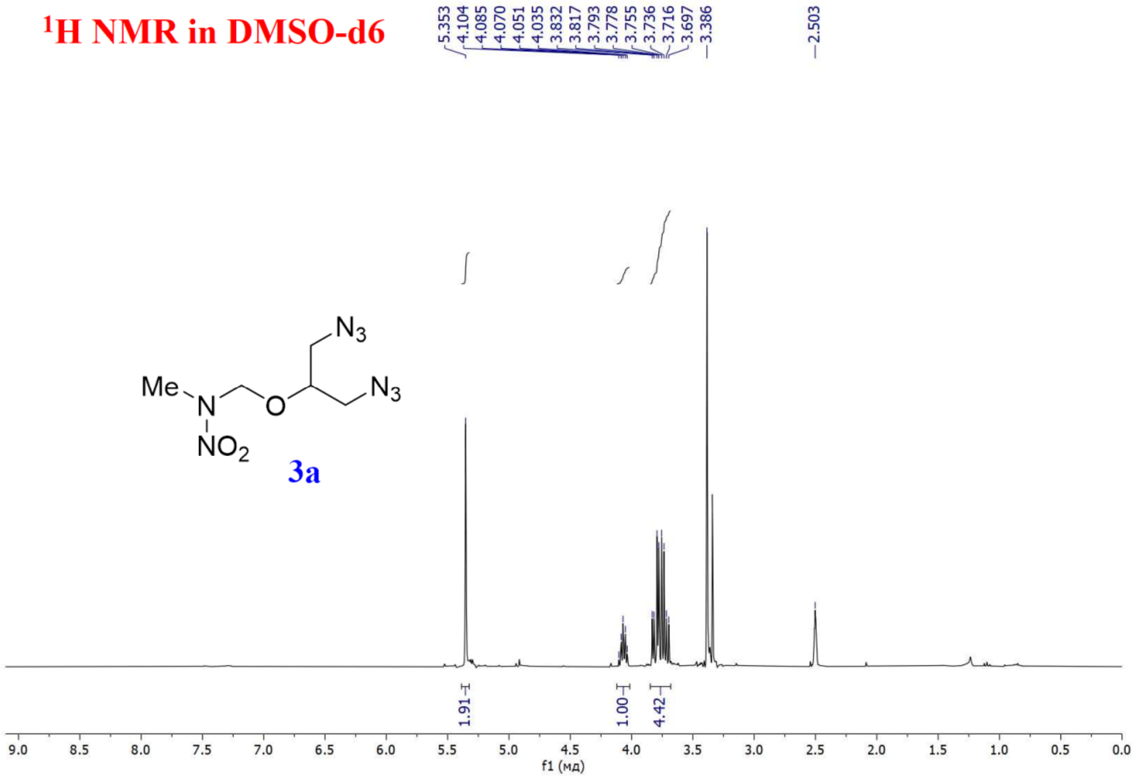

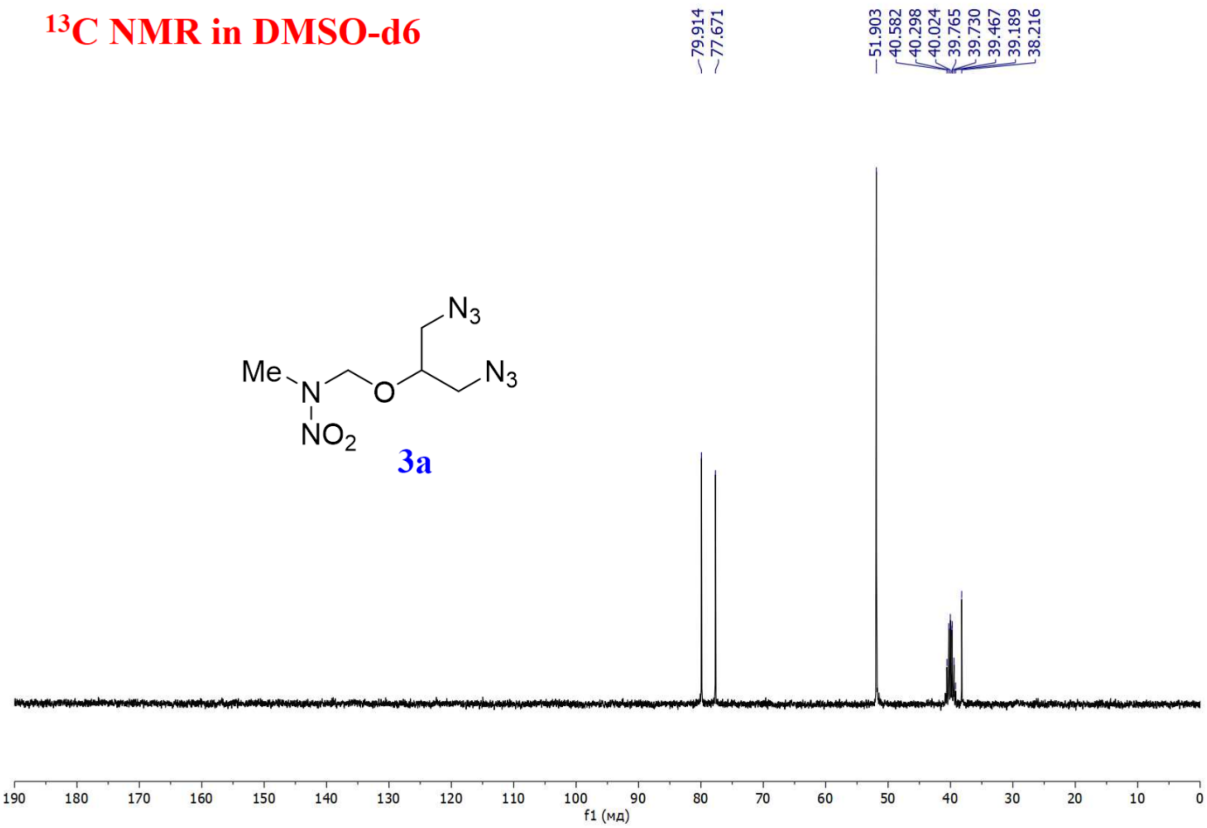

^{13}C NMR in DMSO-d6
79.914
77.671
51.903
40.582
40.298
40.024
39.765
39.730
39.467
39.189
38.216
Me
N
NO$_2$
O
N$_3$
N$_3$
3a
190 180 170 160 150 140 130 120 110 100 90 80 70 60 50 40 30 20 10 0
f1 (мд)

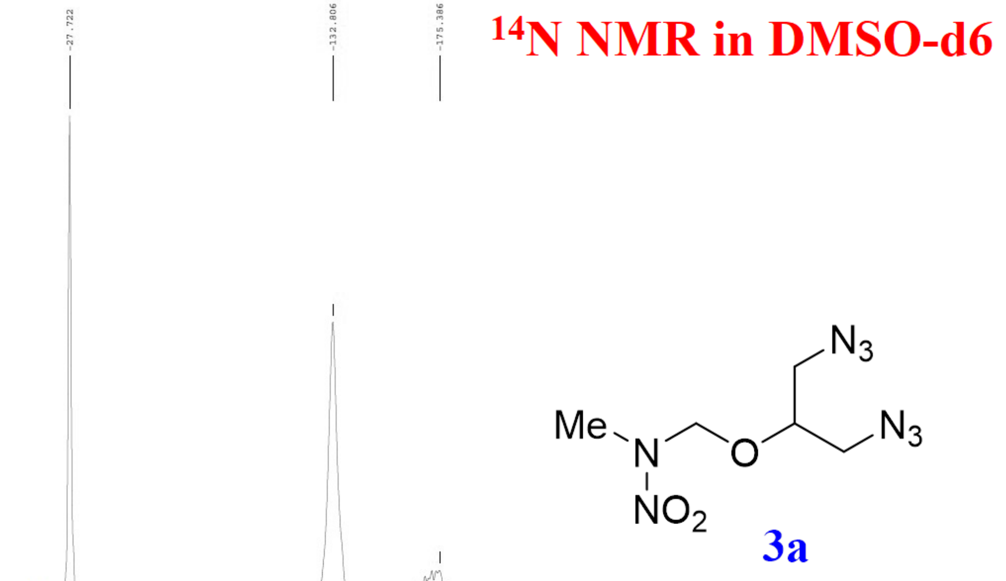

^{14}N NMR in DMSO-d6
-27.722
-132.806
-175.386
Me
N
NO$_2$
O
N$_3$
N$_3$
3a
-50 -100 -150 -200 -250 -300 -350 ppm

^{1}H NMR in DMSO-d6

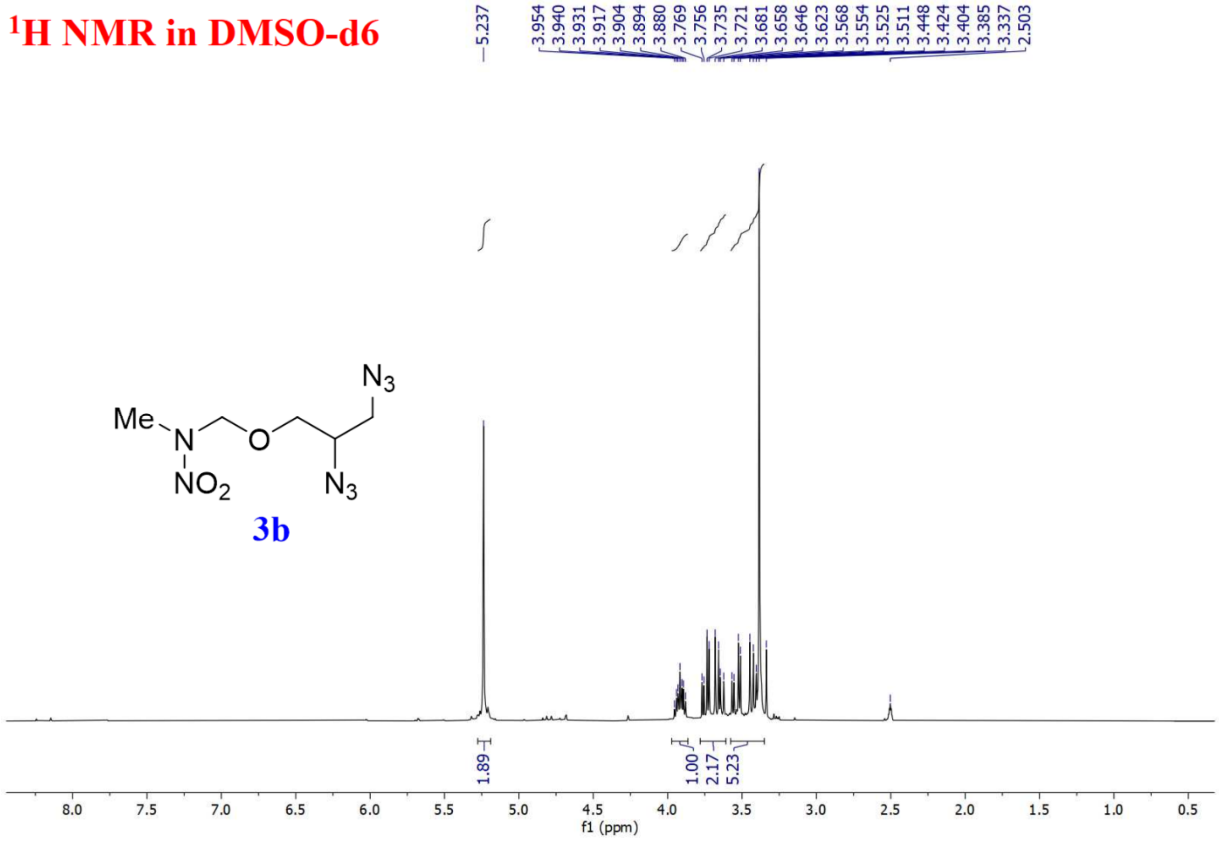

^{13}C NMR in DMSO-d6

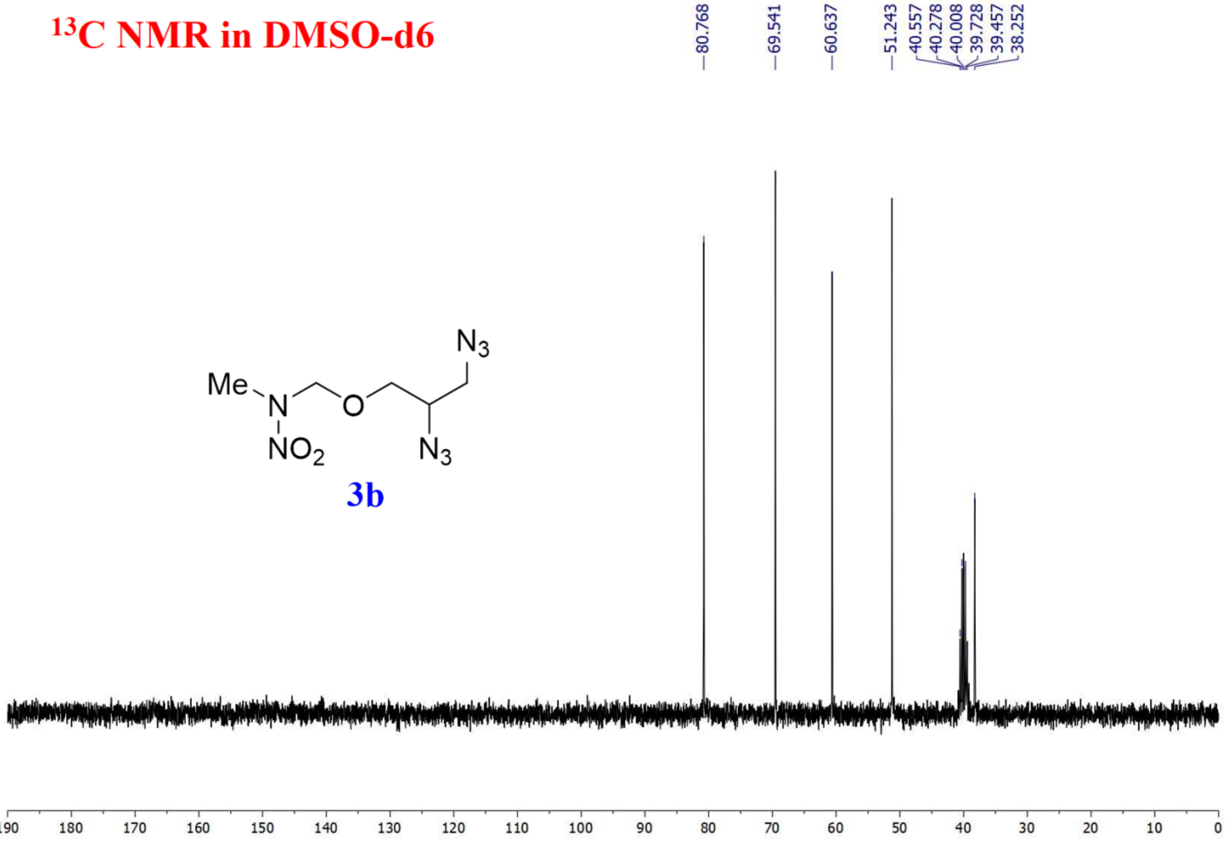

^{14}N NMR in DMSO-d6

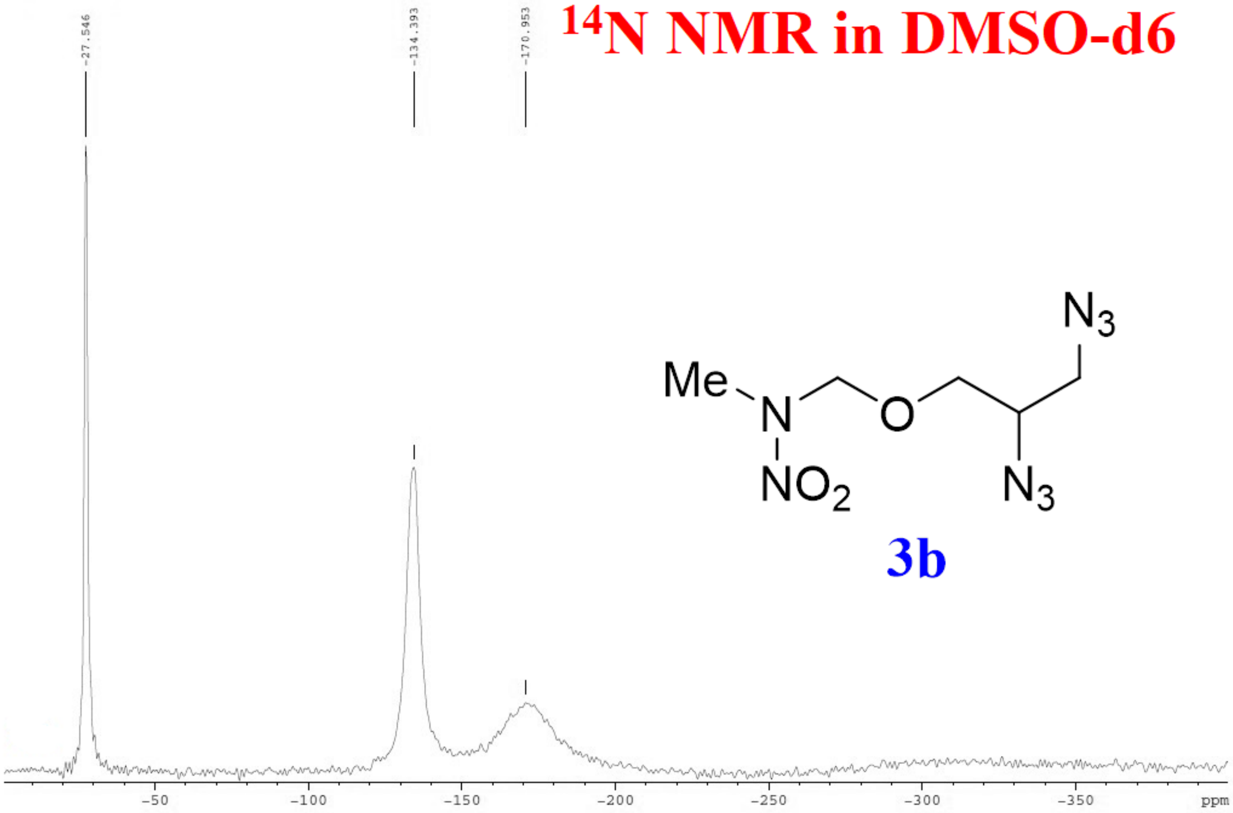

^{1}H NMR in DMSO-d6

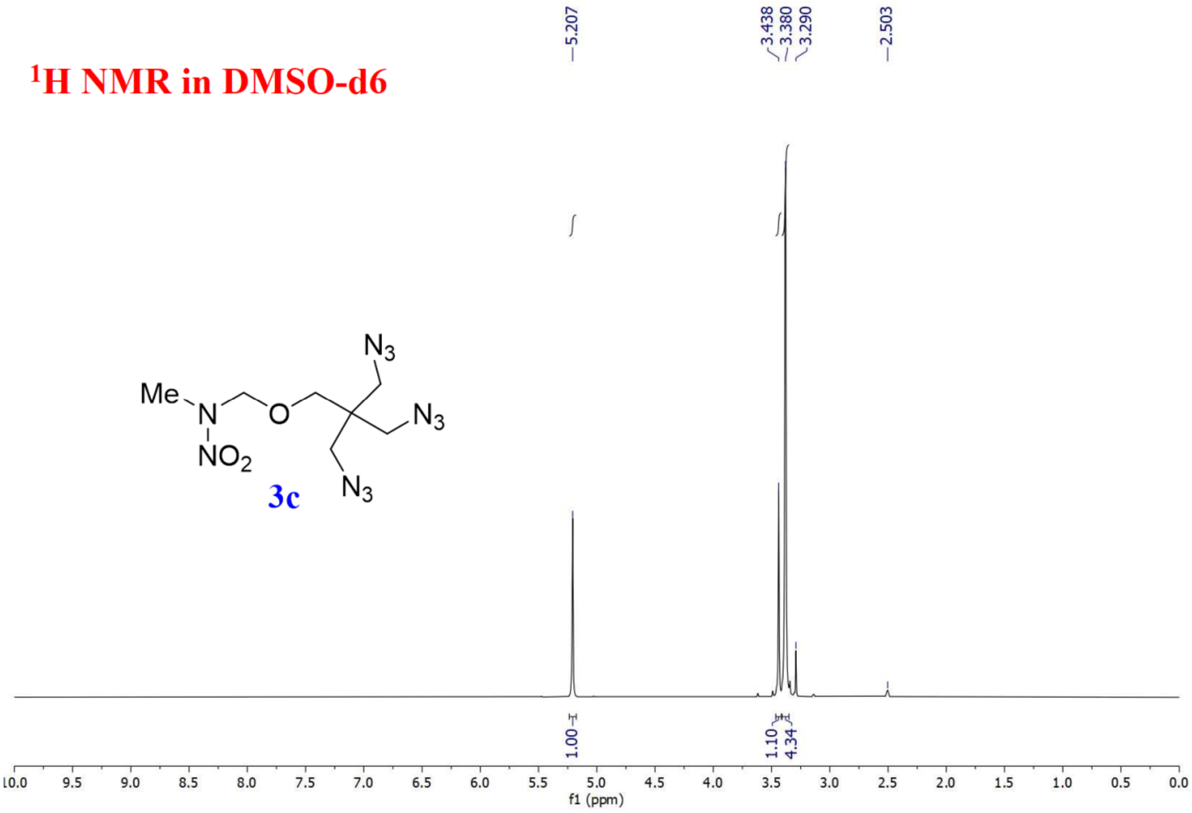

^{13}C NMR in DMSO-d6

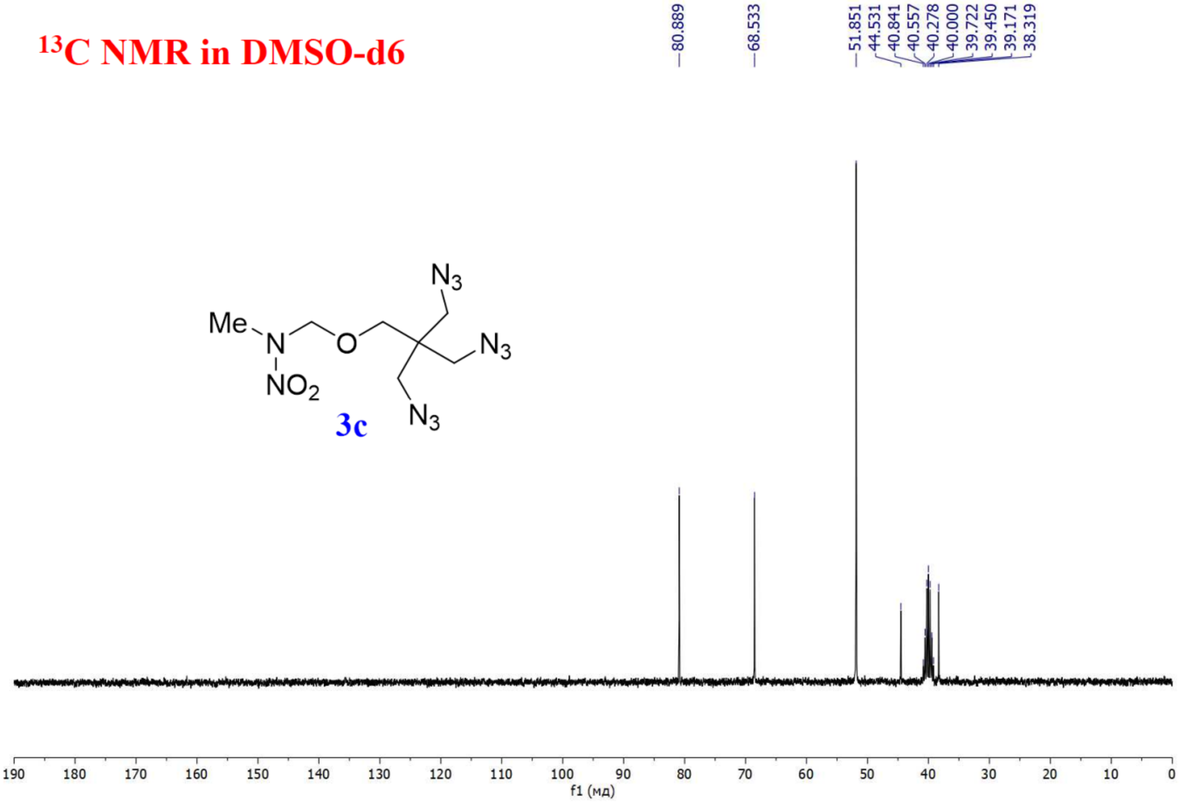

^{14}N NMR in DMSO-d6

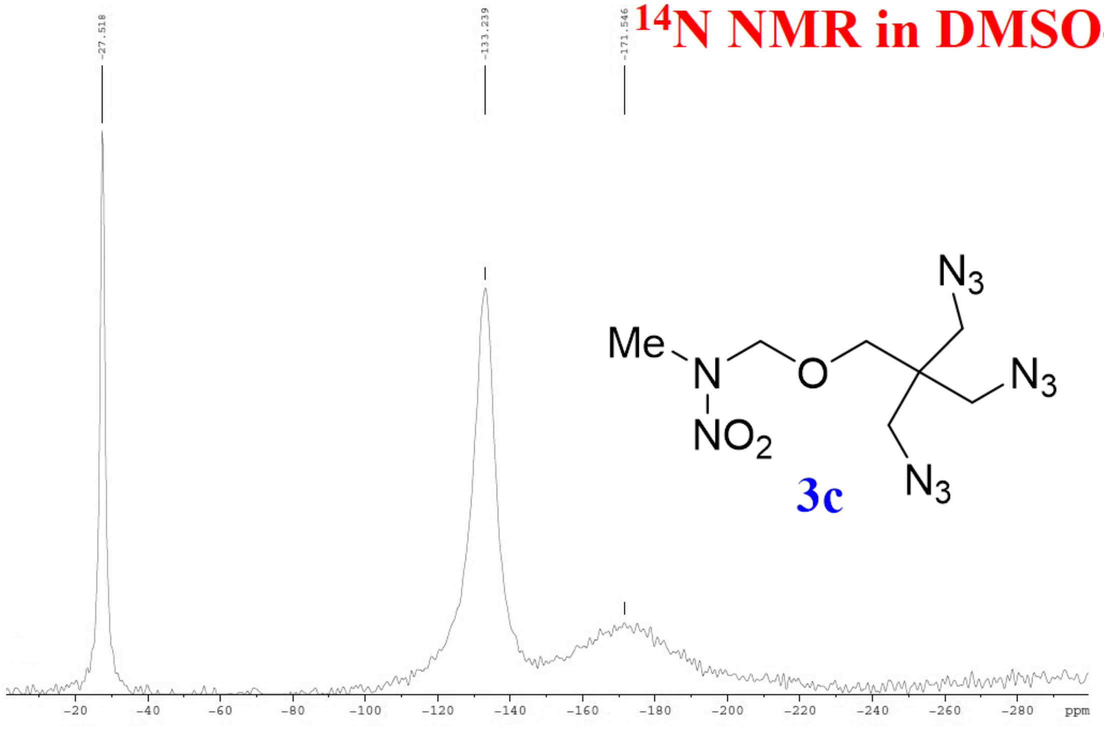

^{1}H NMR in DMSO-d6

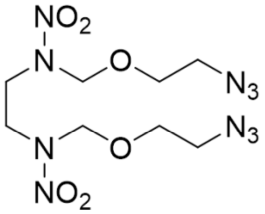

3d

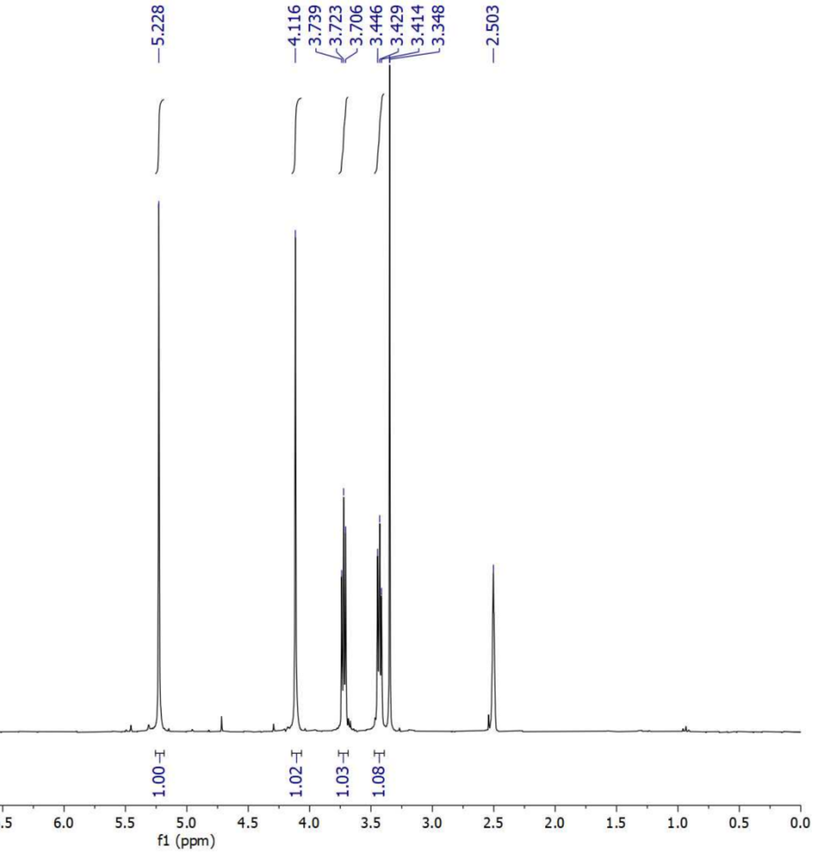

^{13}C NMR in DMSO-d6

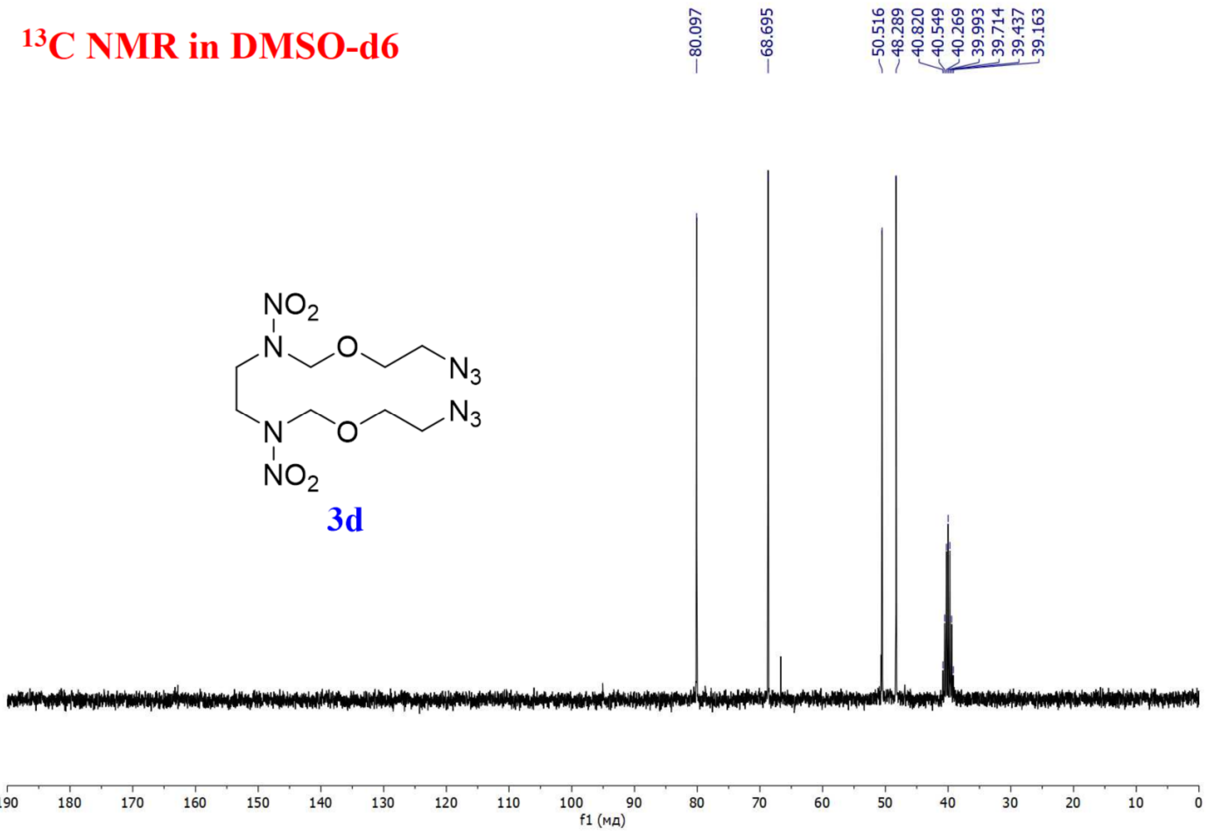

^{14}N NMR in DMSO-d6

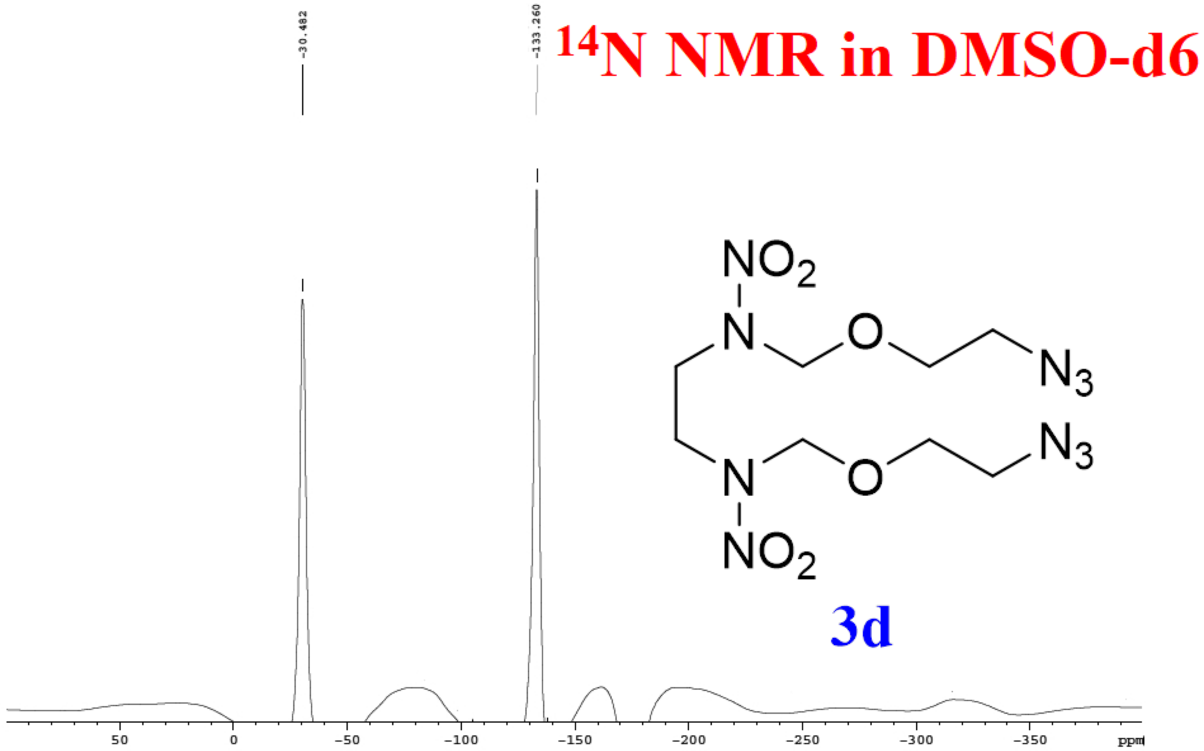

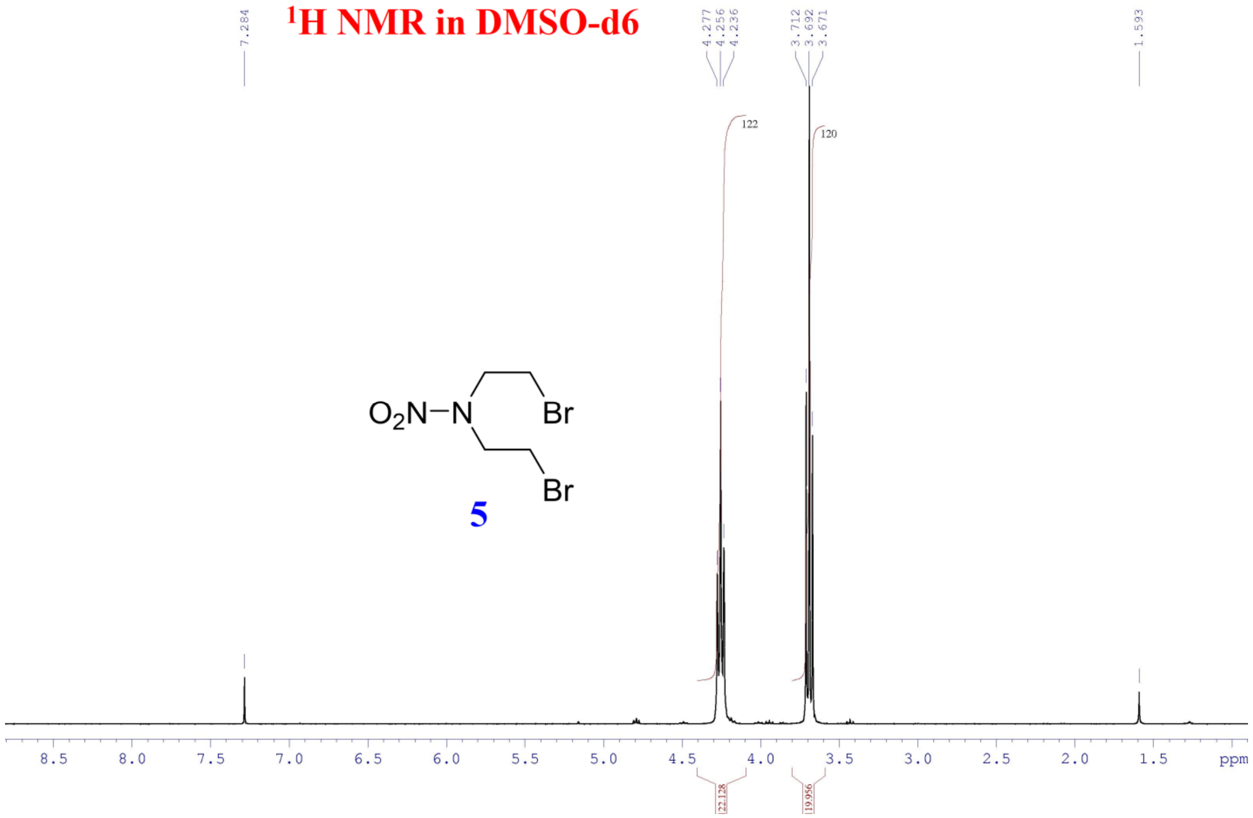

^{1}H NMR in DMSO-d6
7.284
4.277
4.256
4.236
3.712
3.692
3.671
1.593
122
120
O_2N-N
Br
Br
5
8.5 8.0 7.5 7.0 6.5 6.0 5.5 5.0 4.5 4.0 3.5 3.0 2.5 2.0 1.5 ppm
22.128
19.956

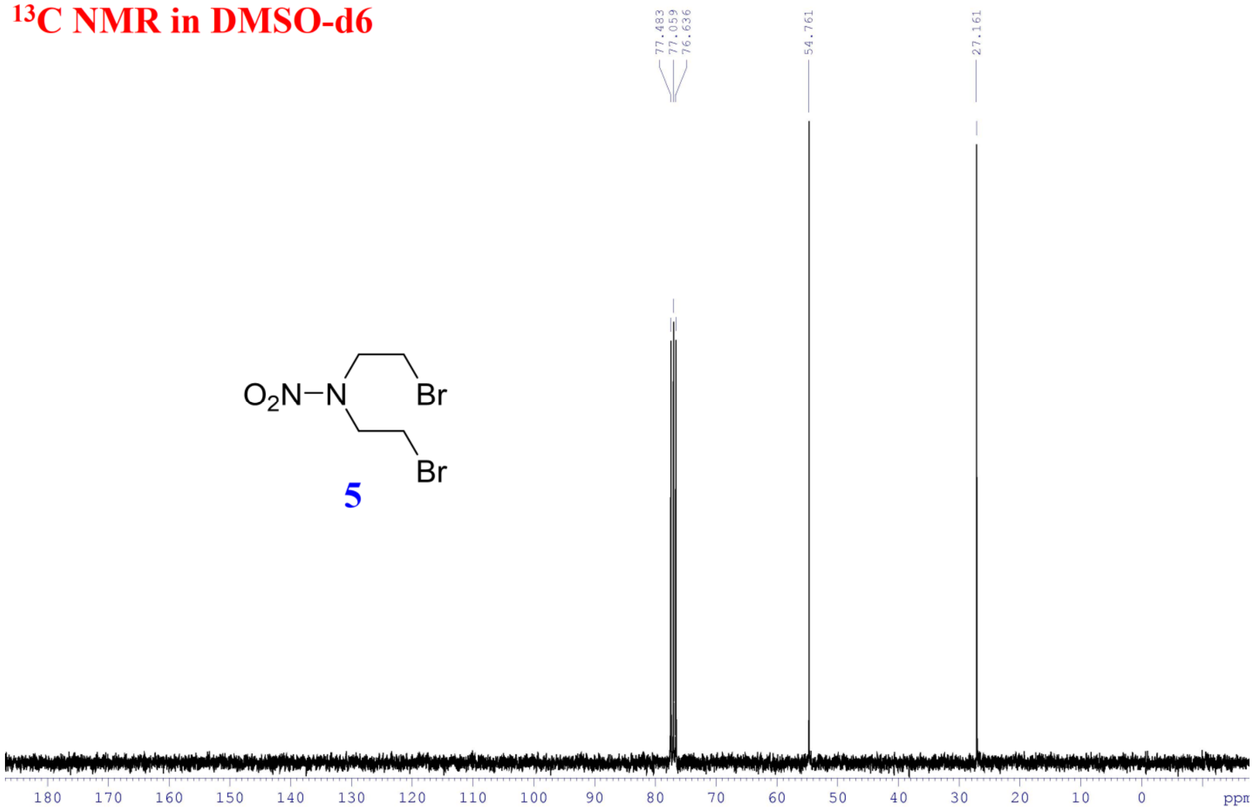

^{13}C NMR in DMSO-d6
77.483
77.059
76.636
54.761
27.161
O_2N-N
Br
Br
5
180 170 160 150 140 130 120 110 100 90 80 70 60 50 40 30 20 10 0 ppm

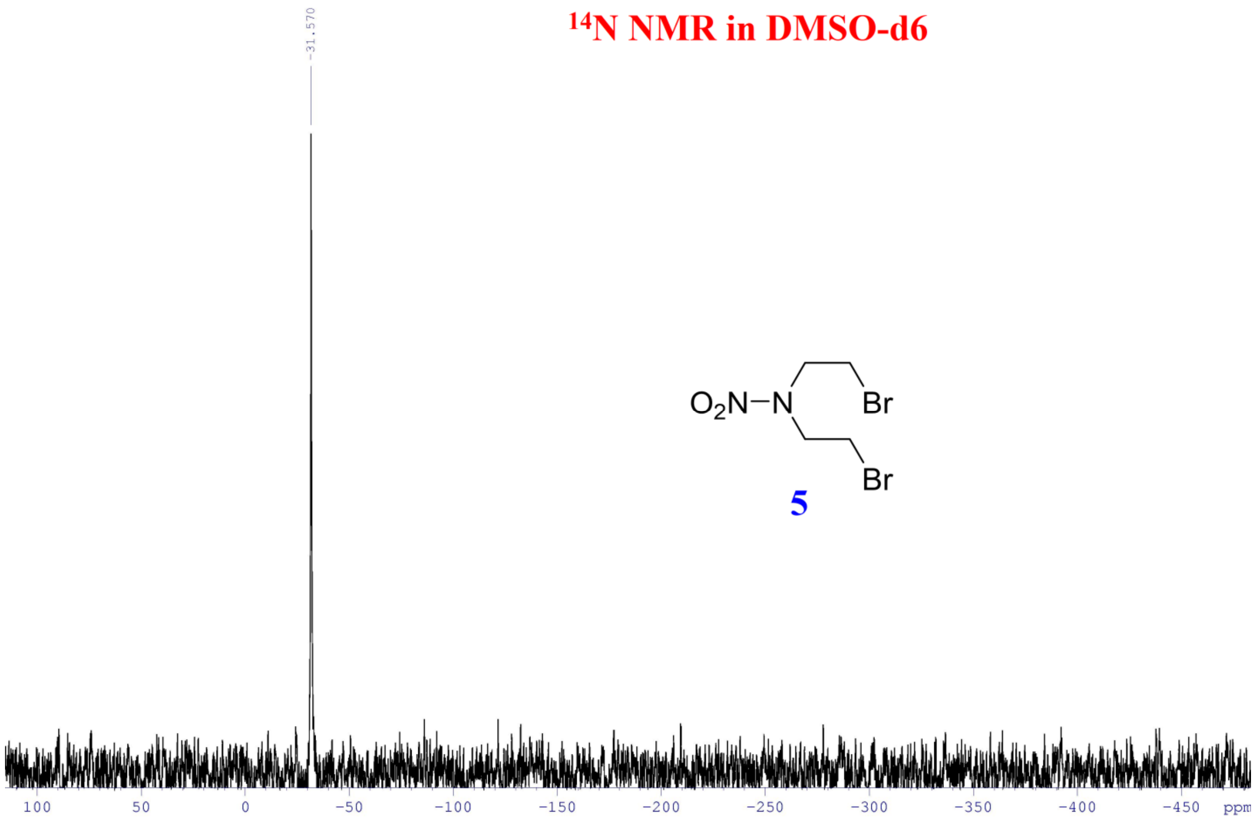

^{14}N NMR in DMSO-d6
-31.570
O_2N-N
Br
Br
5
100
50
0
-50
-100
-150
-200
-250
-300
-350
-400
-450
ppm

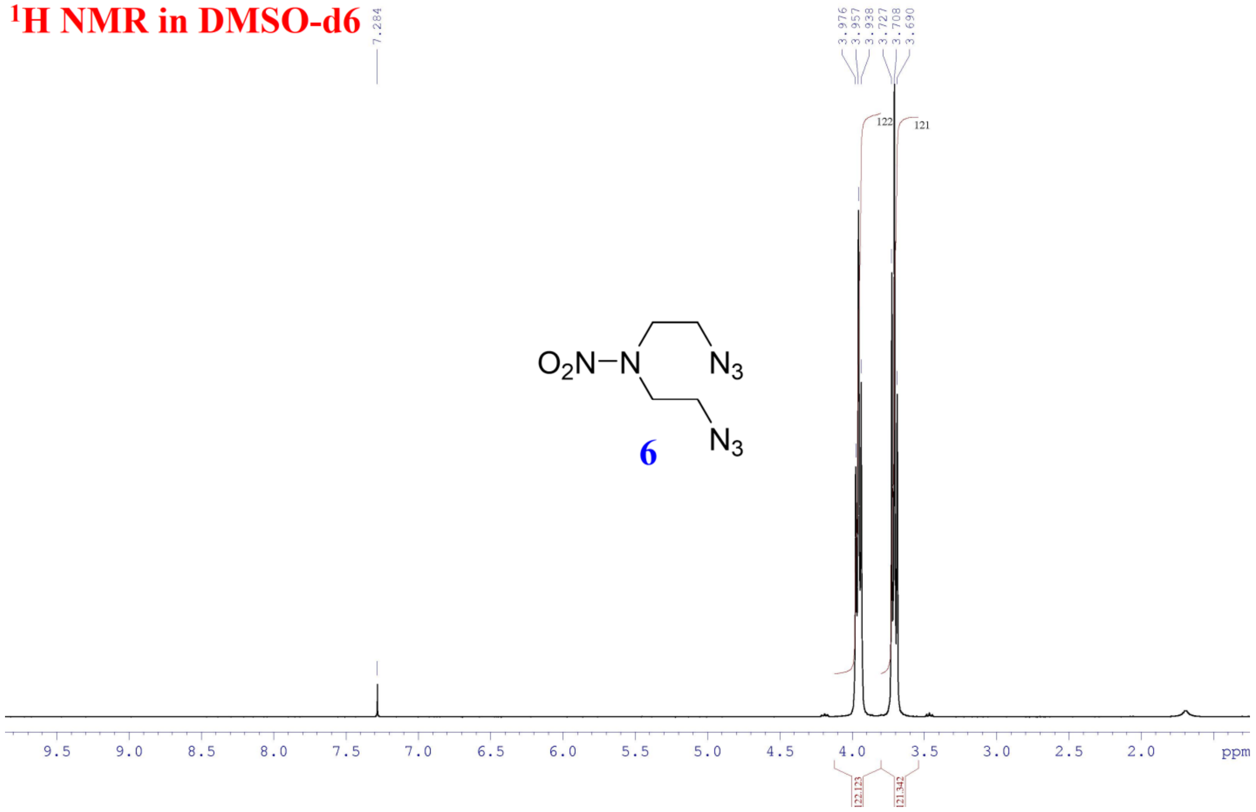

^{1}H NMR in DMSO-d6
7.284
3.976
3.957
3.938
3.727
3.708
3.690
122
121
O_2N-N
N_3
N_3
6
9.5
9.0
8.5
8.0
7.5
7.0
6.5
6.0
5.5
5.0
4.5
4.0
3.5
3.0
2.5
2.0
ppm
122.123
121.342

^{13}C NMR in DMSO-d6

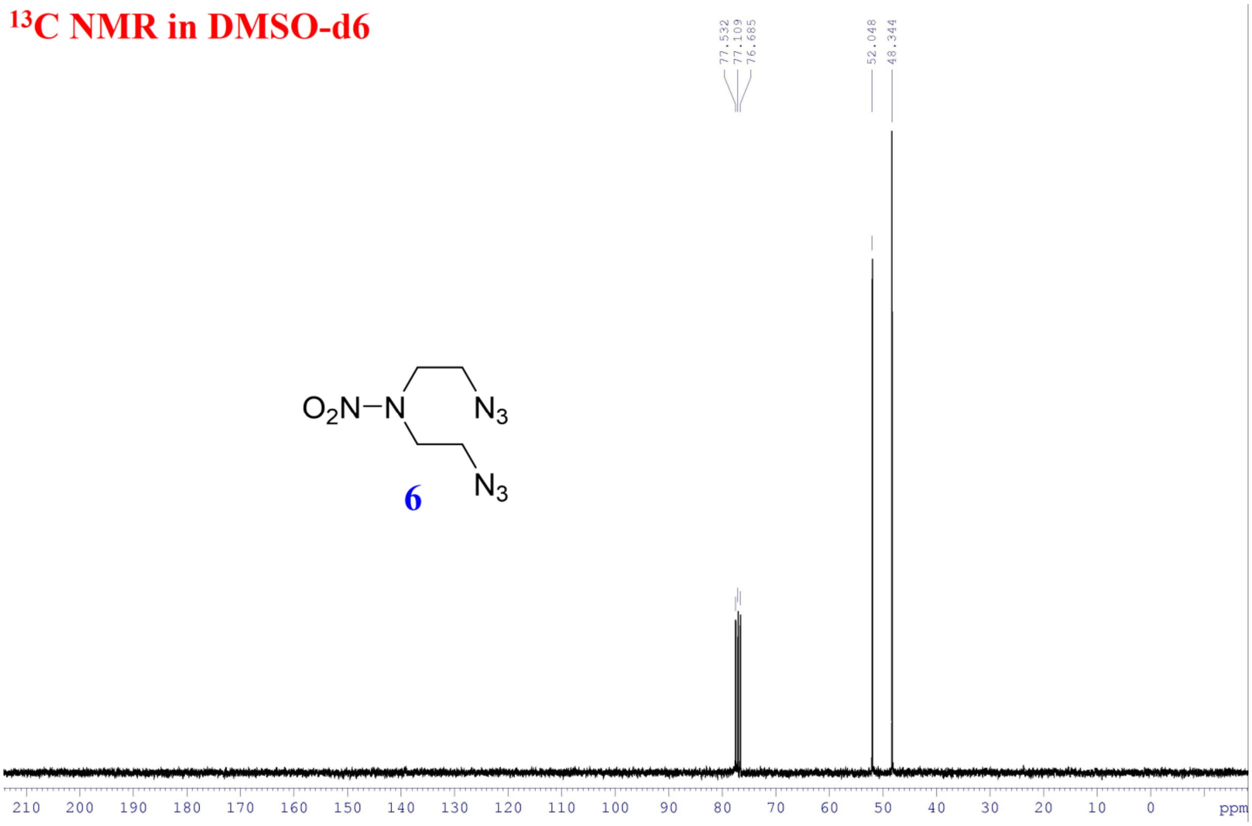

^{14}N NMR in DMSO-d6

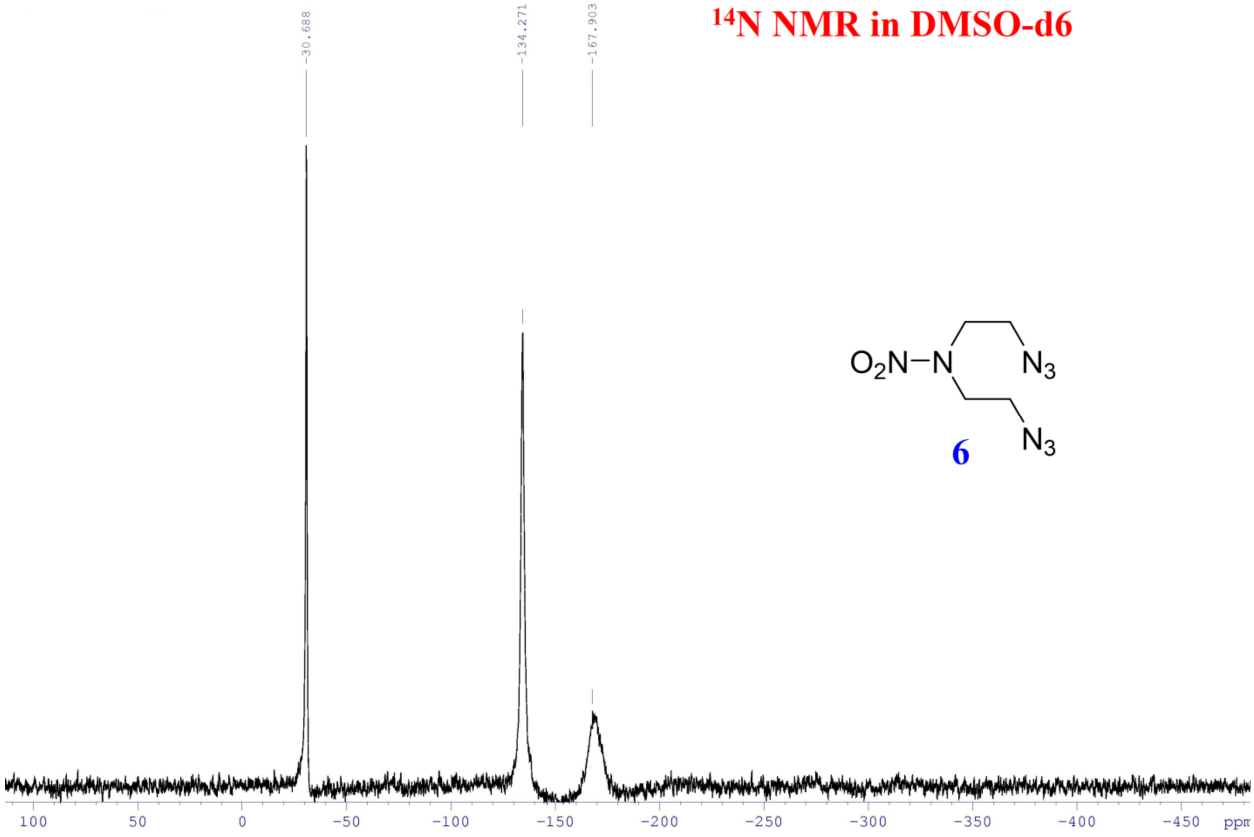

^{1}H NMR in DMSO-d6

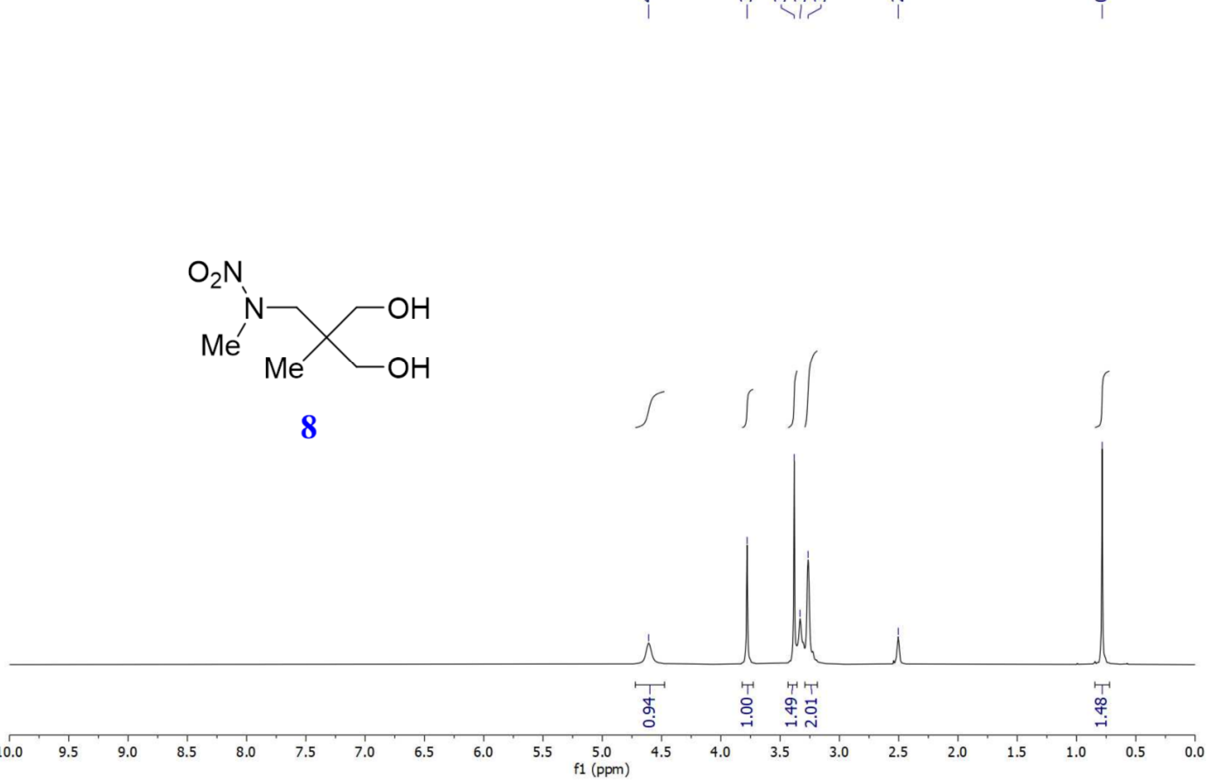

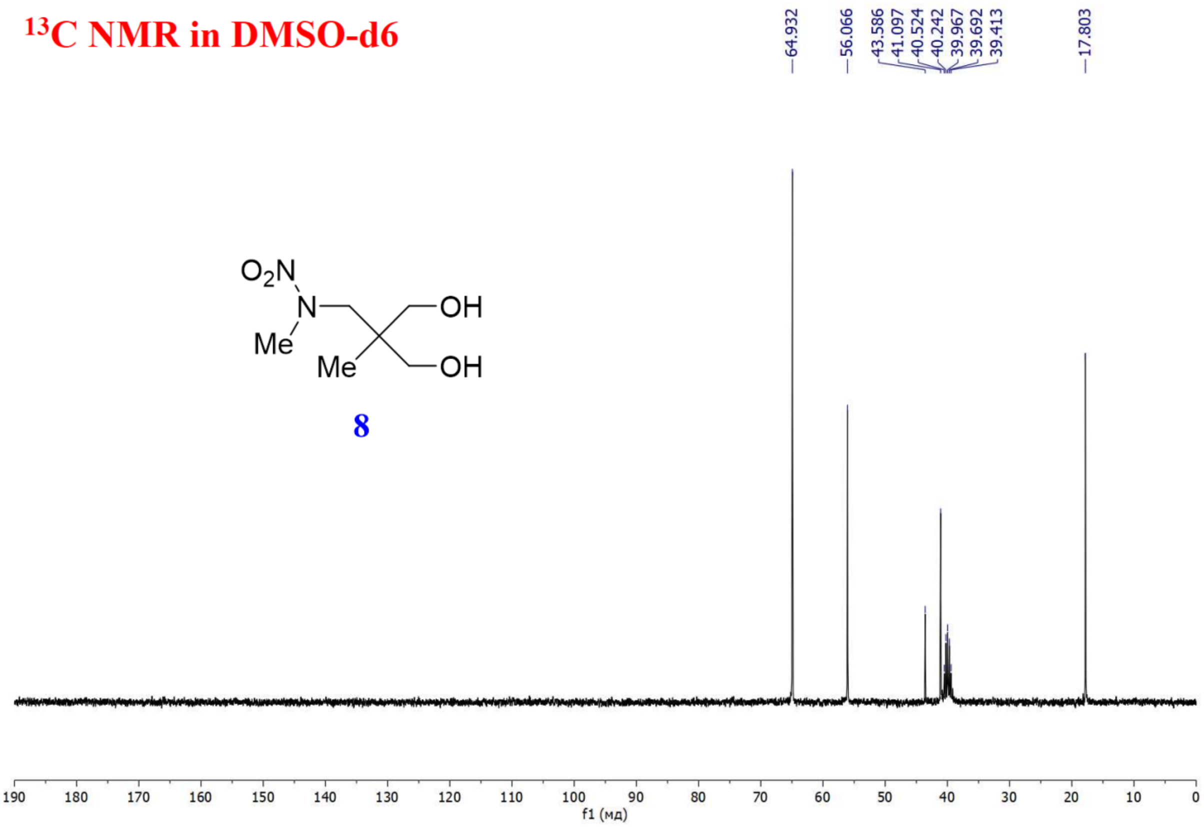
13C NMR in DMSO-d6
64.932
56.066
43.586
41.097
40.524
40.242
39.967
39.692
39.413
17.803
O2N
N
Me
Me
OH
OH
8
190 180 170 160 150 140 130 120 110 100 90 80 70 60 50 40 30 20 10 0
f1 (мд)

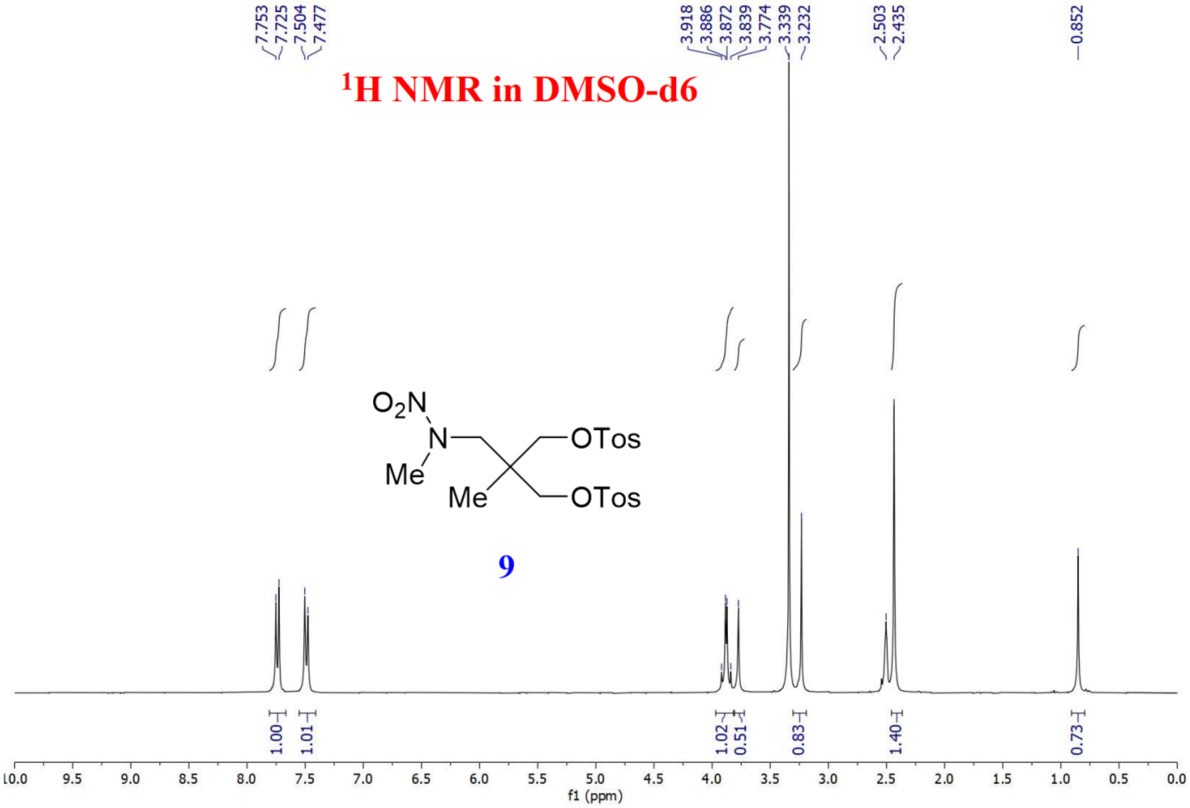
7.753
7.725
7.504
7.477
3.918
3.886
3.872
3.839
3.774
3.339
3.232
2.503
2.435
0.852
1H NMR in DMSO-d6
O2N
N
Me
Me
OTos
OTos
9
1.00
1.01
1.02
0.51
0.83
1.40
0.73
10.0 9.5 9.0 8.5 8.0 7.5 7.0 6.5 6.0 5.5 5.0 4.5 4.0 3.5 3.0 2.5 2.0 1.5 1.0 0.5 0.0
f1 (ppm)

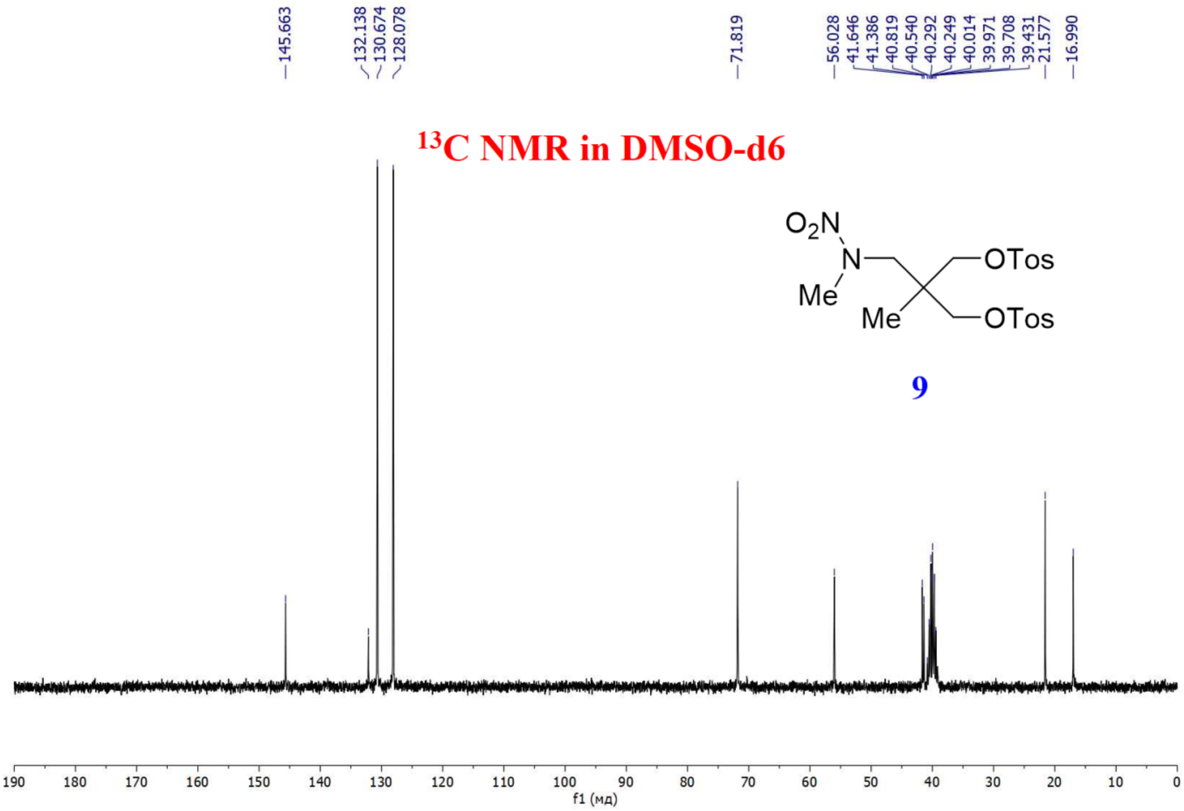
145.663
132.138
130.674
128.078
71.819
56.028
41.646
41.386
40.819
40.540
40.292
40.249
40.014
39.971
39.708
39.431
21.577
16.990
^{13}C NMR in DMSO-d6
O_2N
N
Me
Me
OTos
OTos
9
190 180 170 160 150 140 130 120 110 100 90 80 70 60 50 40 30 20 10 0
f1 (мд)

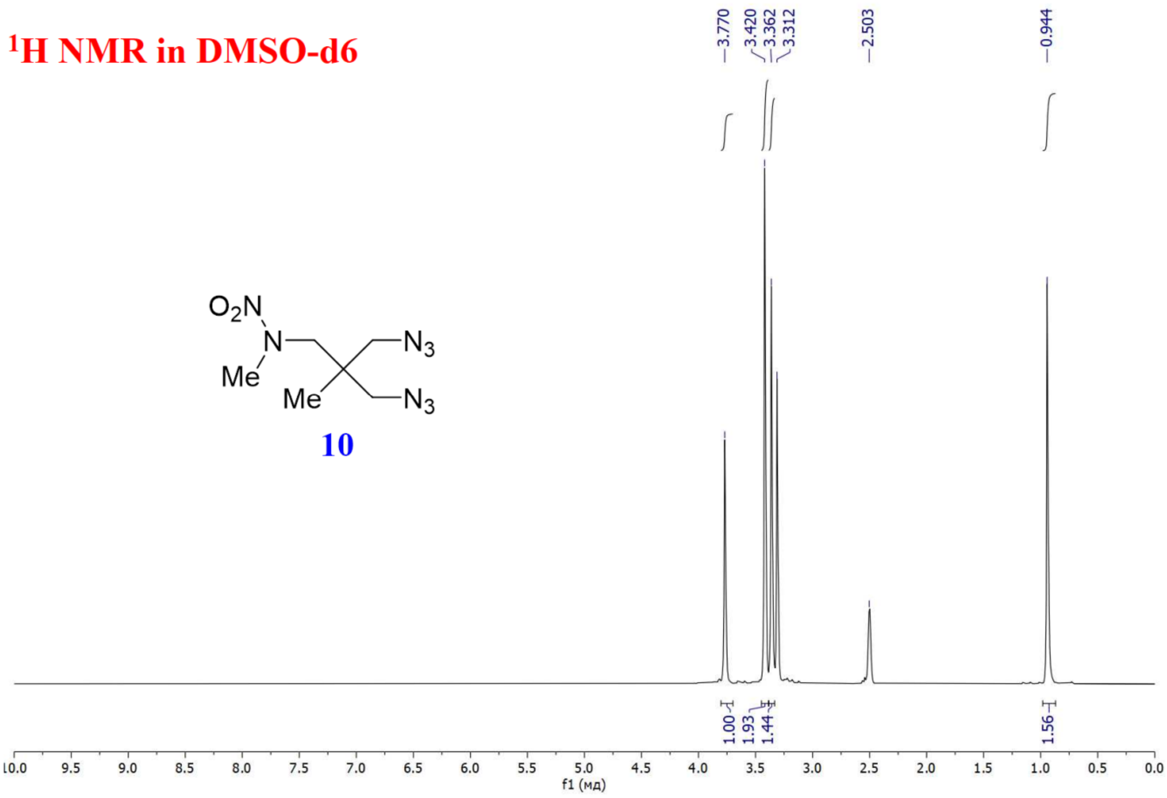
^{1}H NMR in DMSO-d6
3.770
3.420
3.362
3.312
2.503
0.944
O_2N
N
Me
Me
N_3
N_3
10
1.00
1.93
1.44
1.56
10.0 9.5 9.0 8.5 8.0 7.5 7.0 6.5 6.0 5.5 5.0 4.5 4.0 3.5 3.0 2.5 2.0 1.5 1.0 0.5 0.0
f1 (мд)

^{13}C NMR in DMSO-d6

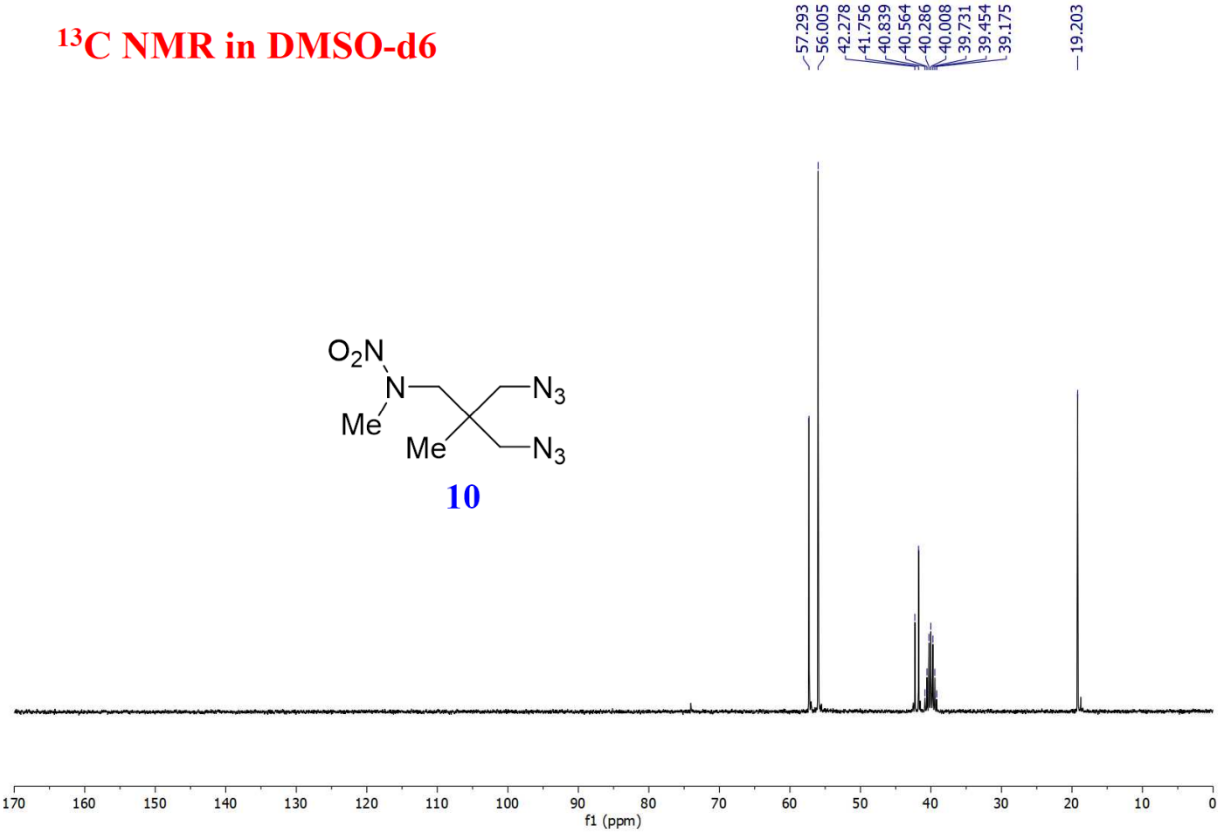

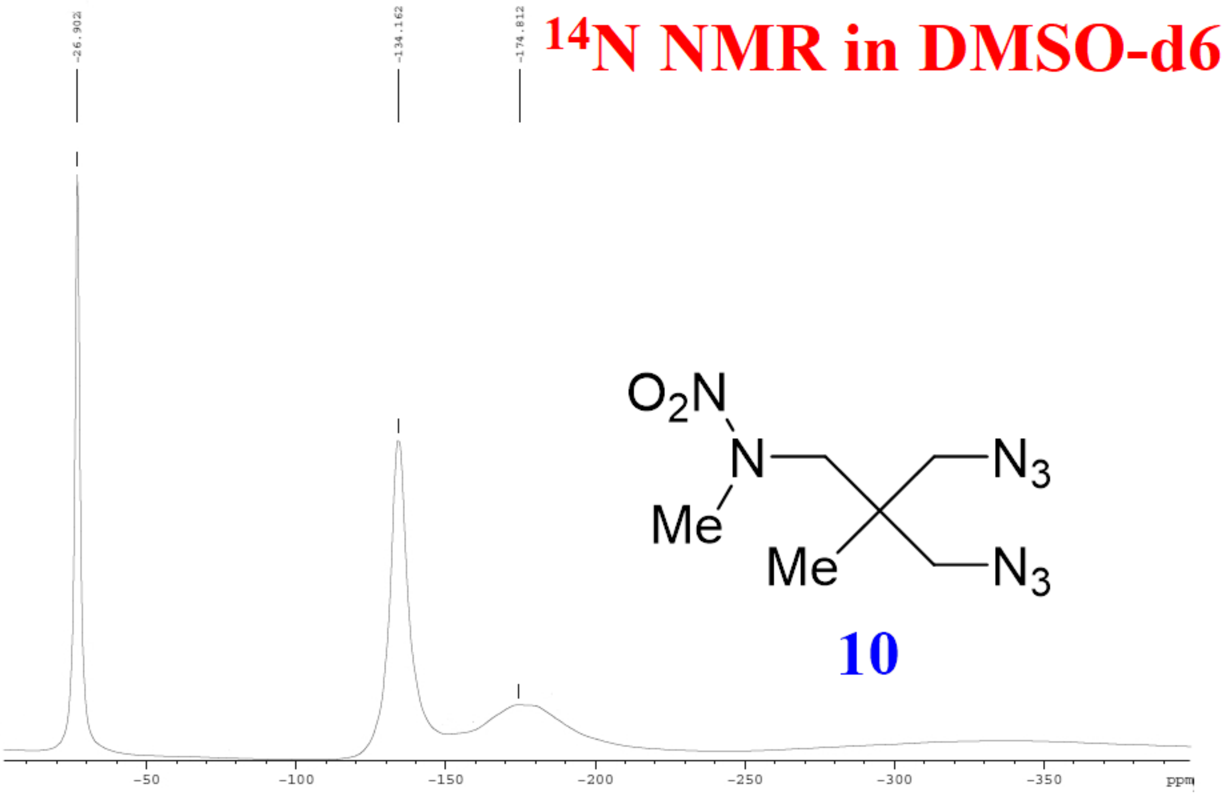

14N NMR in DMSO-d6
-26.902
-134.162
-174.812
O2N
N
Me
Me
N3
N3
10
-50
-100
-150
-200
-250
-300
-350
ppm

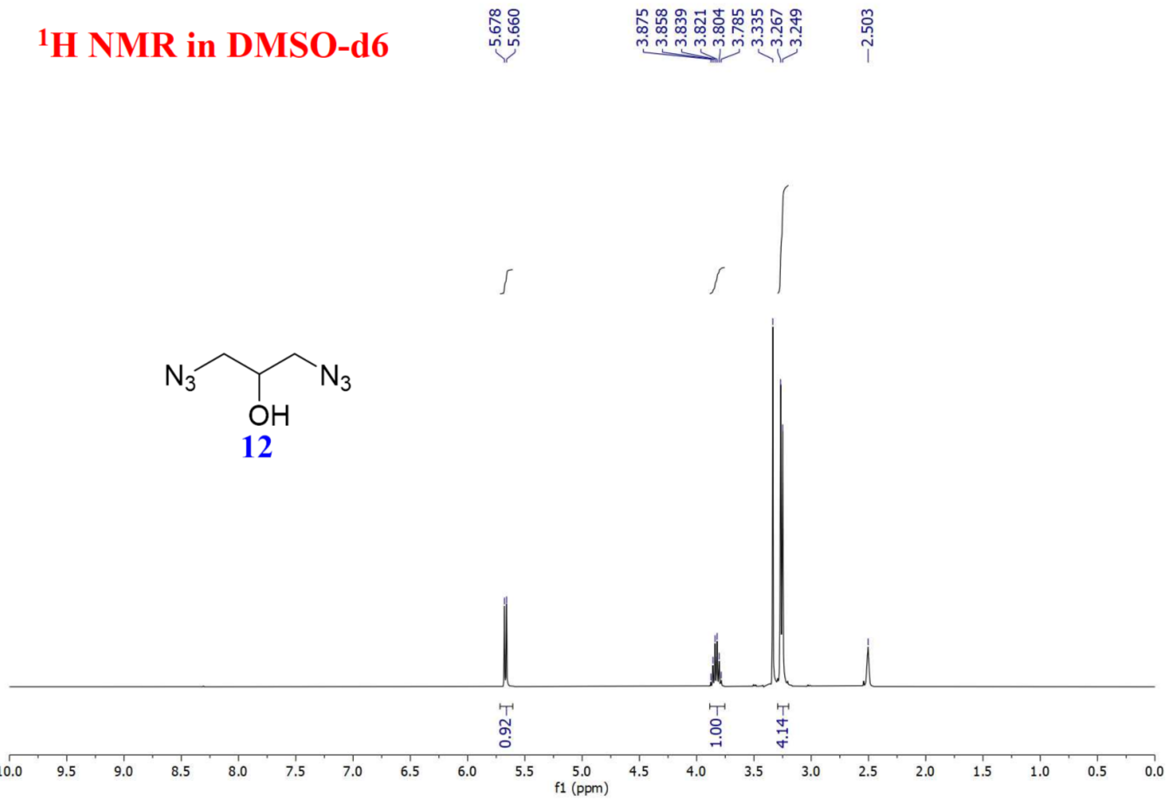

1H NMR in DMSO-d6
5.678
5.660
3.875
3.858
3.839
3.821
3.804
3.785
3.335
3.267
3.249
2.503
N3
N3
OH
12
0.92
1.00
4.14
10.0
9.5
9.0
8.5
8.0
7.5
7.0
6.5
6.0
5.5
5.0
4.5
4.0
3.5
3.0
2.5
2.0
1.5
1.0
0.5
0.0
f1 (ppm)

^{1}H NMR in DMSO-d6

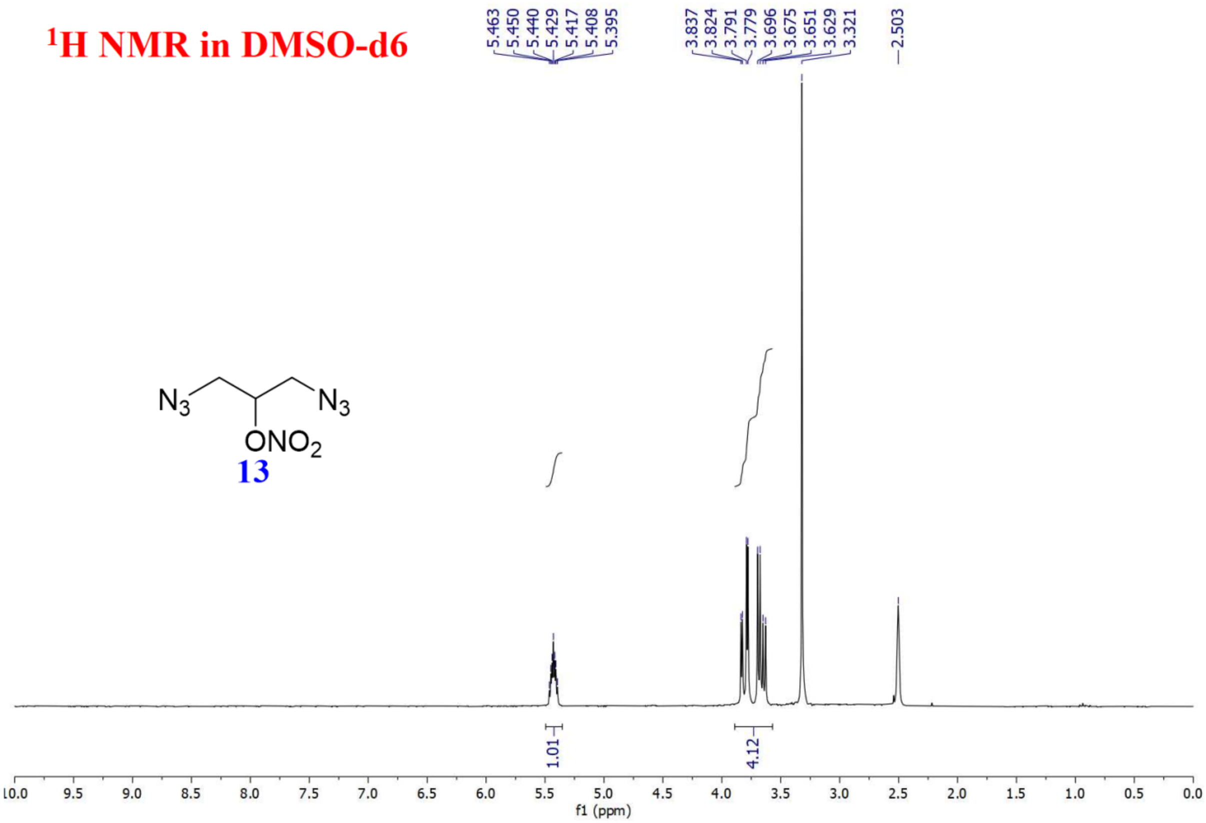

13
